# Bioengineered polyester beads co-displaying protein and carbohydrate-based antigens induce protective immunity against bacterial infection

**DOI:** 10.1038/s41598-018-20205-7

**Published:** 2018-01-30

**Authors:** Majela González-Miró, Laura M. Rodríguez-Noda, Mildrey Fariñas-Medina, Barbara Cedré-Marrero, Sandra Madariaga-Zarza, Caridad Zayas-Vignier, Mabel Hernández-Cedeño, Torsten Kleffmann, Dagmar García-Rivera, Vicente Vérez-Bencomo, Bernd H. A. Rehm

**Affiliations:** 1 Institute of Fundamental Sciences, Massey University, Palmerston North, New Zealand; 2 Finlay Vaccine Institute, La Havana, Cuba; 3 Biochemistry Department, University of Otago, Otago, New Zealand; 4 Centre for Cell Factories and Biopolymers, Griffith Institute for Drug Discovery, Griffith University, Nathan, Queensland Australia

## Abstract

The efficacy of protein and carbohydrate antigens as vaccines can be improved via particulate delivery strategies. Here, protein and carbohydrate antigens used in formulations of vaccines against *Neisseria menigitidis* were displayed on *in vivo* assembled polyester beads using a combined bioengineering and conjugation approach. An endotoxin-free mutant of *Escherichia coli* was engineered to produce translational fusions of antigens (*Neisseria* adhesin A (NadA) and factor H binding protein (fHbp) derived from serogroup B) to the polyhydroxybutyrate synthase (PhaC), in order to intracellularly assemble polyester beads displaying the respective antigens. Purified beads displaying NadA showed enhanced immunogenicity compared to soluble NadA. Both soluble and particulate NadA elicited functional antibodies with bactericidal activity associated with protective immunity. To expand the antigen repertoire and to design a more broadly protective vaccine, NadA-PhaC beads were additionally conjugated to the capsular polysaccharide from serogroup C. Co-delivery of surface displayed NadA and the capsular polysaccharide induced a strong and specific Th1/Th17 mediated immune response associated with functional bactericidal antibodies. Our findings provide the foundation for the design of multivalent antigen-coated polyester beads as suitable carriers for protein and polysaccharide antigens in order to induce protective immunity.

## Introduction

There is a growing world-wide demand for efficient and broadly protective vaccines for the prevention of infectious diseases. Although safe and specific, subunit vaccines often lack immunogenicity and, due to the limited antigen repertoire, show impaired capacity to induce broadly protective immunity^1^. Here we explored a combined bioengineering and chemical conjugation strategy to produce self-assembled particulate vaccines co-displaying protein and carbohydrate antigens in order to overcome the weaknesses of subunit vaccines.

As an example, we chose to design a vaccine against *Neisseria meningitidis* which can cause severe meningitis often resulting in permanent disability in survivors of the disease^2^. Broadly protective and cost-effectively produced vaccines are on demand. The WHO reported a global incidence of ~1.3 million cases per annum with a mortality of 5–15% and more than 25% of disabled survivors^2^. The pathogenicity of this bacterium is based on virulence factors such as capsular polysaccharides (CPS), outer membranes proteins (adhesins and porins) and lipopolysaccharides. Additionally, pathogenicity is enhanced by evasion of the host immune defense mechanisms via antigen mimicry and sequestration of factor H, a regulator of complement activation^3^. The CPS diversity results in currently 13 serogroups. Six serogroups A, B, C, W135, Y and X were found to be virulent, causing disease^4,5^. As emerging multiple antibiotic resistances impair antibiotic treatment outcomes, prevention by vaccination is now the preferred strategy.

To obtain a good memory response in the target population^6^, conjugation of different CPS to a carrier proteins (conjugate vaccines) provided an efficient strategy to prevent this disease. Tetravalent conjugated vaccines such as Menactra^®^ (Sanofi Pasteur), Menveo^®^ (Novartis) and Nimenrix^®^ (GSK) are used to prevent meningococcal diseases from serogroups A, C, W135 and Y^7^. However, serogroup C is still responsible for many deaths^8^ and serogroup B which causes more than half of the cases of the meningococcal disease world-wide, is not included in any conjugate vaccine as its CPS is similar to polysaccharides presented in human neurologic tissues^9^. Hence subunit vaccines against *N. meningitidis* serogroup B such as VAMENGO-BC^®^ are focused on surface proteins such as outer membrane proteins (OMPs, PorA and PorB)^10–12^. The efficiency of these vaccines is strain specific, limiting their use in many countries^13^. Reverse vaccinology approaches screening the entire genome sequence of *N. meningitidis* serogroup B strain MC58 for immunodominant antigens accelerated the development of protein-based vaccines against this bacterium^14,15^. Relevant proteins such as *Neisseria* adhesin (NadA), factor H binding protein (fHbp), *Neisseria* heparin binding antigen (NHBA), Genome-derived antigen (GNA) 2091 and GNA 1030 were identified by this technique^16–19^. The above mentioned proteins are included in the recently licensed vaccines 4CMenB Bexsero^®^ (Novartis, 2015) and Trumenba^®^ (Pfizer, 2014)^20^. Recombinant subunit vaccines often present limitations such as the inability to elicit broadly protective immune responses and the necessity of adjuvant in the vaccine formulation in order to boost immunogenicity^21^. Particulate antigen delivery systems hold the promise to overcome these hurdles^22^.

Polymer particles incorporating antigens as a delivery system were found to stabilise antigens, enhance uptake of antigen by antigen presenting cells (APCs) and provide an antigen depot effect for an extended display of antigens^23,24^. Naturally formed bacterial inclusions made of a polyhydroxybutyrate (PHB) core surrounded by the PHB synthase (PhaC) have been considered for bioengineering of particulate vaccines. Translational fusions of protein antigens to PhaC enabled display of respective antigens on the surface of the PHB beads which elicited strong and specific immune responses^25–29^. PHB beads were successfully bioengineered to display antigens from intracellular pathogens like *Mycobacterium tuberculosis* and the Hepatitis C virus. These particulate vaccine candidates elicited both Th1 and Th2 immune responses^26,30,31^. The elicited immune responses mediated protective immunity against both diseases^26^. Antigen-displaying PHB beads were also produced in other bacterial hosts such as *Mycobacterium smegmatis* and *Lactococcus lactis*^25,32^. In a recent study, the bacterial pathogen’s own polyhydroxyalkanoate inclusion assembly was engineered to produce antigen-displaying particulate vaccines^33^.

The aim of this study was to design and produce a multivalent antigen delivery system co-displaying protein and carbohydrate-based antigens as alternative vaccine formulation for induction of protective immunity against infectious diseases such as caused by *N. meningitidis*. Hence, we first explored the possibility of designing and producing proteinaceous antigen-displaying PHB inclusions. Secondly, we aimed to conjugate the CPS from *N.meningitidis* serogroup C to antigen coated PHB beads^8,34,35^. The respective antigen-coated PHB beads were subjected to physicochemical and immunological characterization.

## Results

### Bioengineering of *Escherichia coli* for production of antigen-displaying PHB inclusions and soluble antigens

**Figure 1:**
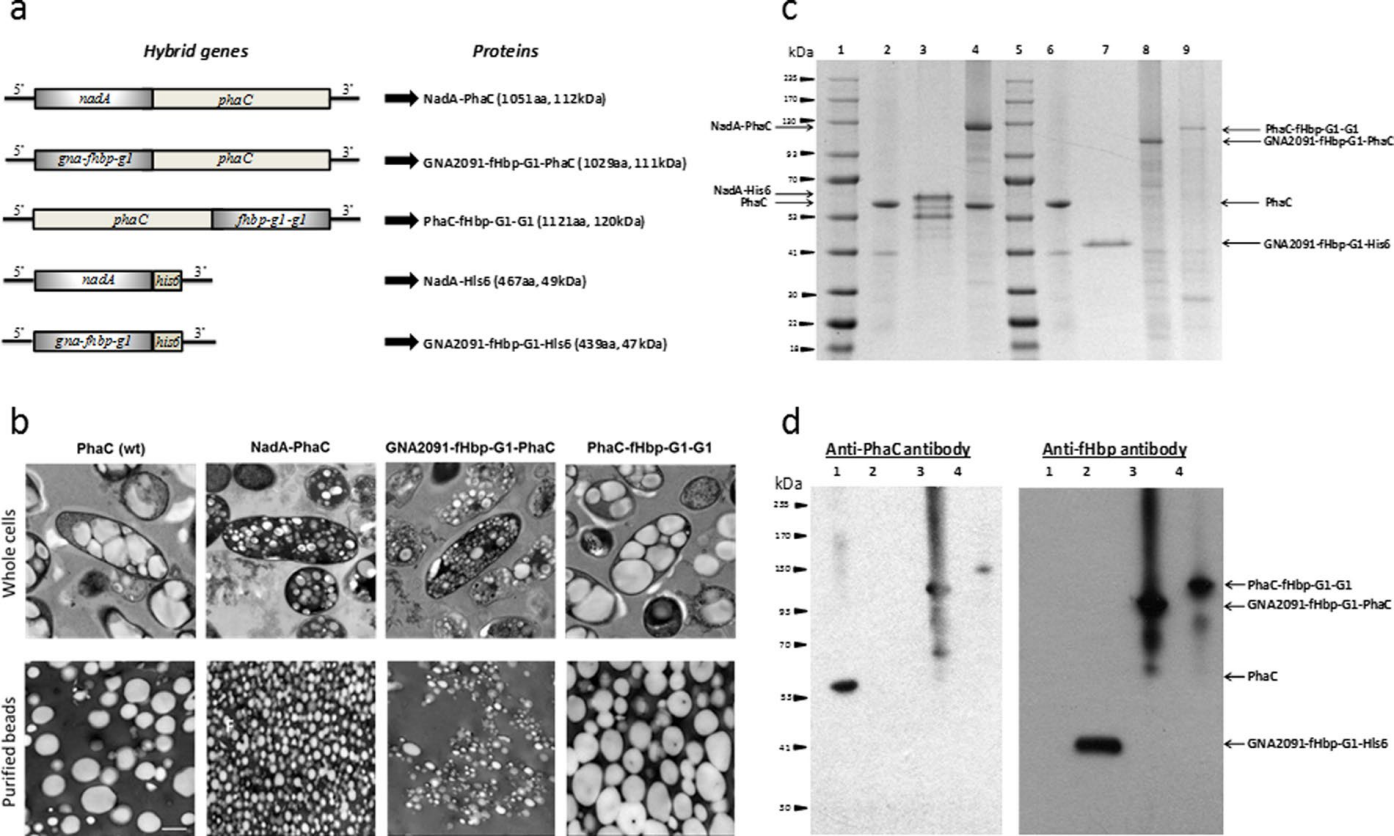
Biological production and characterization of antigen coated PHB beads. (**a**) Schematic representation of the hybrid genes mediating formation of antigen displaying PHB beads or production of the respective soluble antigen. (**b**) PHB inclusion production and beads size evaluation by Transmission Electron Microscopy (TEM) using whole cells of recombinant *E. coli* (Clearcoli^TM^) and the respective purified PHB beads; (**c**) SDS-PAGE analysis of the protein profile of isolated PHB beads as well as the purified His-tagged proteins (selected antigens). Lane 1, MW (molecular weight) standard, (GangNam-Stain, iNtRON BIOTECHNOLOGY); Lane 2, non-antigen displaying PHB beads (PhaC ~64 kDa); Lane 3, NadA-His6 protein ~50 kDa; Lane 4, NadA-PhaC fusion protein (~113 kDa) on PHB beads; Lane 5, MW standard, (GangNam-Stain, iNtRON BIOTECHNOLOGY); Lane 6, non-antigen displaying PHB beads (PhaC ~64 kDa); Lane 7, GNA2091-fHbp-G1-His6 protein (~47 kDa); Lane 8, GNA2091-fHbp-G1-PhaC fusion protein (~111 kDa) on PHB beads; Lane 9, fHbp-G1-G1-PhaC fusion protein (~120 kDa) on PHB beads. (**d**) Immunoblotting using commercial monoclonal anti-fHbp antibodies (JAR4, NIBCS, UK) and monospecific polyclonal anti-PhaC antibodies were performed to further confirm the identity of the respective fusion proteins. Fusion proteins with the anticipated MW containing fHbp were detected. Lane 1, PhaC protein, (non-antigen displaying PHB beads); Lane 2, GNA2091-fHbp-G1-His6 protein; Lane 3, GNA2091-fHbp-G1-PhaC fusion protein on PHB bead; Lane 4, fHbp-G1-G1-PhaC fusion protein on PHB beads.

Five plasmids were constructed to either mediate formation of antigen-displaying PHB beads or production of the respective soluble antigen (Fig. 1a).

*Clearcoli*^*TM*^, an endotoxin-free mutant of *E. coli*, harbouring plasmid pMCS69 encoding PhaA and PhaB, which sequentially catalyze the synthesis of PHB precursors, was transformed with plasmids encoding PhaC fusion proteins (Supplementary Table 1). TEM images of cells harbouring plasmids encoding PhaC fusion proteins showed the presence of discrete spherical inclusions indicative of PhaC functionality (Fig. 1b). Interestingly, cells harbouring plasmids encoding NadA- and GNA2091-fHbp-G1-containing PhaC fusion proteins mediated formation of smaller PHB inclusions when compared to the other PHA granule-forming strains (Fig. 1b).

SDS-PAGE analysis of the protein profile of the isolated PHB beads as well the purified His6-tagged proteins indicated successful production of the respective proteins (Fig. 1c). The molecular identity of these proteins was confirmed by tryptic peptide fingerprinting analysis using MALDI-TOF/MS (Supplementary Table 2). In the case of fHbp variants, additional immunoblotting and ELISA were performed (Fig. 1d, Supplementary Fig. 1).

**Figure 2:**
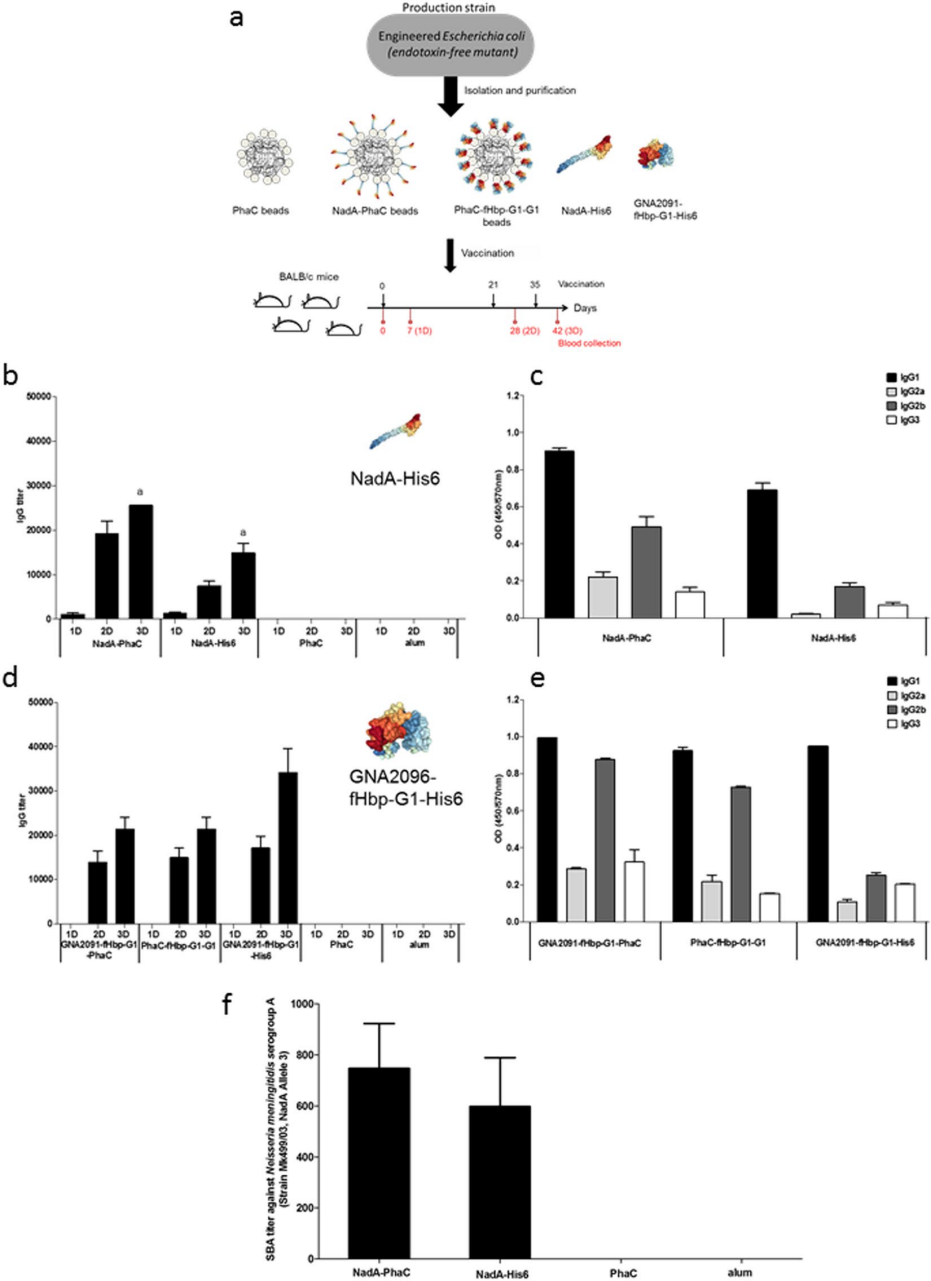
PHB bead production and their immunogenicity. (**a**) Schematic representation of the PHB beads production, composition and immunization schedule. Depicted protein structures are derived from the protein data bank as follows: NadA variant 5 (4CJD), factor H binding protein (mutant G1) (2Y7S). PHB, polyhydroxybutyrate; (**b**) Assessment of antibodies binding to NadA; **c**) Assessment of NadA-specific IgG subclass titers using pooled sera from the 6 animals; (**d**) Assessment of antibodies binding to GNA2091-fHbp-G1 specific; (**e**) Assessment of GNA2091-fHbp-G1 specific IgG subclass titers using pooled sera from the 6 animals; (**f**) Serum bactericidal activity (SBA) evaluationis. Statistical significance with (*p* < 0.01) was determined by Mann-Whitney test and is indicated by letter in Fig. 2a. No statistical significance with (*p* < 0.05) was determined by Mann-Whitney test, Fig. 2d IgG between groups vaccinated with GNA2091-fHbp-G1-PhaC beads and GNA2091-fHbp-G1-His6.

As the surface charge of antigen beads might impact immunogenicity, the Zeta potential of the PHB beads was determinate as a function of pH (3–7.5). Negative Zeta potentials at all measured pH values suggested an anionic surface charge for all beads (Supplementary Table 3). In addition, the amount of the neisserial antigens attached to the PHB beads was determined as previously described^36^ and used to adjust the amount of antigen per dose to be used for the animal trial (Supplementary Table 4). Purified proteinaceous antigen-displaying PHB beads and soluble antigen were then subjected to immunogenicity studies by vaccinating mice (Fig. 2a).

### Immunological properties of antigen displaying PHB beads

Mice were vaccinated with the various PHB bead suspensions as well as soluble antigen (control) (Table 1). All vaccinated animals remained alive and healthy during the entire study. In the groups immunised with PHB beads small granulomas were observed (up to 2 mm) at the injection site, but no suppuration was detected. Vital organs such as kidneys, lung, liver and spleen from immunized mice showed no differences to those from mice that received the placebo (alum).

**Table 1:** Immunization groups for proteinaceous antigens and features.

Immunization group	NadA amount (µg/mouse)	GNA2091-fHbp-G1 amount (µg/mouse)	fHbp-G1-G1 amount (µg/mouse)	Adjuvant amount (mg/mouse)^84^
NadA-PhaC	2	0	0	0.1
GNA2091-fHbp-G1-PhaC	0	7	0	0.1
PhaC-fHbp-G1-G1	0	0	1	0.1
NadA-His6	2	0	0	0.1
GNA2091-fHbp-G1-His6	7	0	0	0.1
PhaC (7 µg of PhaC/mouse)	0	0	0	0.1
alum	0	0	0	0.1

The antibody responses in sera were assayed seven days after each vaccination using ELISA (Fig. 2a). Assessment of antibodies binding to NadA showed that the mean IgG titers increased for each group after each vaccination, but the highest titers were achieved after the third dose (Fig. 2b). However, as was expected, animals in the non-antigen displaying PHB bead and alum group (placebo) were negative, respectively (Fig. 2b). Groups immunised with NadA-PhaC presented superior IgG titer mean values than the group immunised with NadA-His6, (Fig. 2b). Immunoglobulin (Ig) subclass evaluation showed that IgG1 was predominant and followed by the IgG2b, while NadA displayed on PHB beads induced the strongest response when compared with the respective soluble protein (Fig. 2b,c).

Assessment of antibody levels toward GNA2091-fHbp-G1 showed increasing IgG titers with each dose, while the group of the soluble GNA2091-fHbp-G1 tended to have the highest IgG levels (Fig. 2d). The Ig subclass assay results showed that again IgG1 was predominant followed by IgG2b, mainly in groups vaccinated with PHB beads (Fig. 2e).

To evaluate functionality of induced antibodies as an indication of protection against meningococcal infection, the serum bactericidal assay (SBA) is a powerful tool to assess protective efficacy of these vaccine candidates^37^. Here, the functionality of induced antibodies was analyzed *in vitro* by using sera from mice vaccinated with NadA-PhaC, NadA-His6, PhaC wild type and alum, but also including a heterologous strain, *N. meningitidis* serogroup A (Strain 499/03, positive for NadA, Allele 3)^38^. SBA titers showed that antibodies induced by the vaccine candidates mediated killing of the bacterial pathogen (Fig. 2f). This result demonstrated that vaccination with NadA-PhaC beads promoted the production of high and specific functional antibodies, and hence these beads were used for CPS conjugation experiments.

### Chemical conjugation of capsular polysaccharides to PHB beads

**Figure 3:**
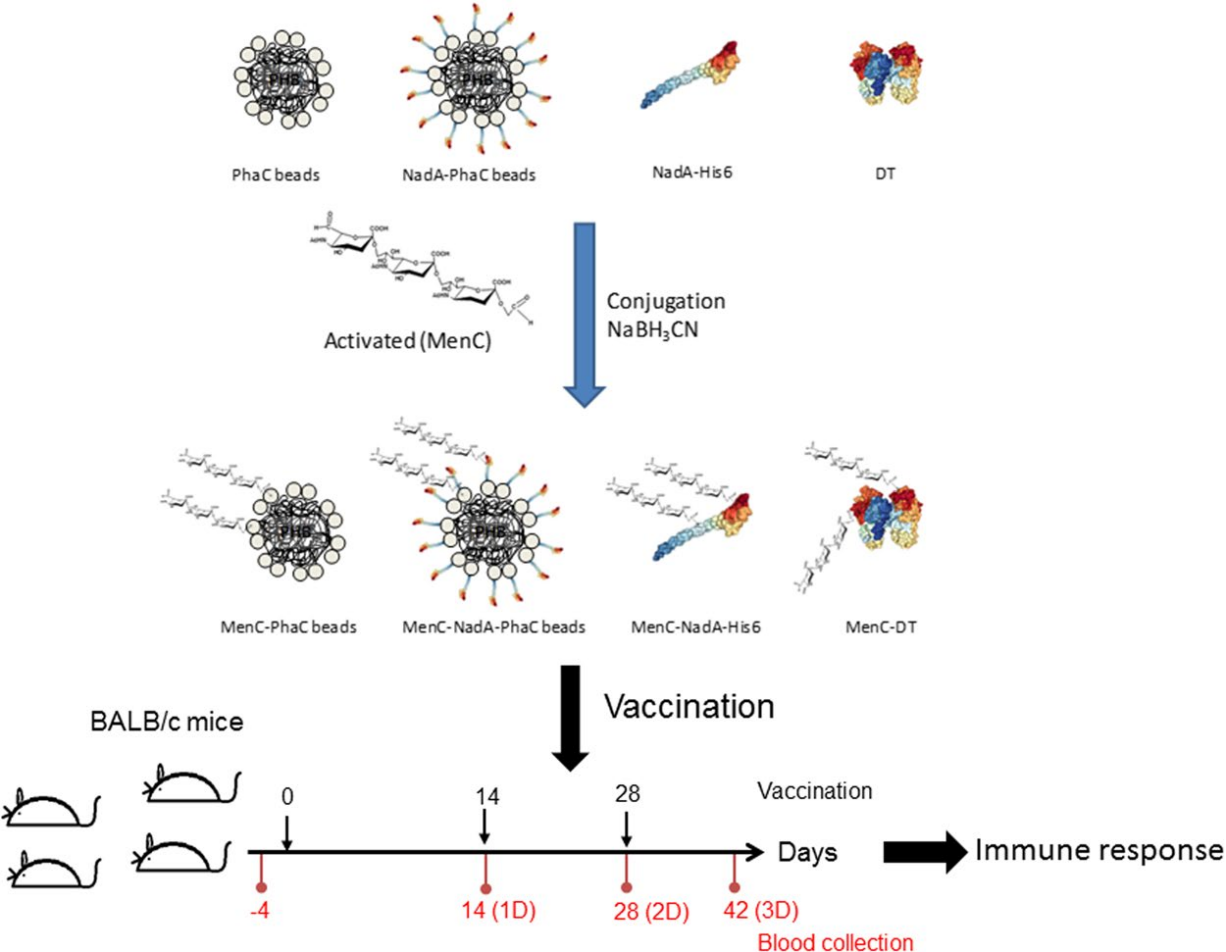
Schematic representation of the chemical conjugation of the CPS (MenC) to soluble and insoluble antigens displayed on PHB beads and immunization schedule. The MenC-TD was included as a classical conjugate control DT, diphtheria toxoid structural model was derived from protein data bank (5I82); NadA (variant 5), protein structure was derived from the protein data bank (4CJD). PHB, polyhydroxybutyrate.

The CPS from *N. meningitidis* serogroup C has been included in all licensed polysaccharide and conjugate vaccines due to its epidemiological relevance. Despite this, serogroup C causes hundreds of deaths among children and adolescents in countries where these vaccines are not accessible^8^. Here, the CPS from *N. meningitidis* serogroup C was subjected to periodic acid oxidation to introduce terminal carbonyl groups. The molecular identity of the activated polysaccharide (APS) was evaluated by ^1^H-NMR spectroscopy (Supplementary Fig. 2). ^1^H-NMR analysis confirmed the preservation of the general chemical structure, while the signal deletions indicated the removal of the O-acetyl groups upon activation reaction as previously shown^39^. Classical reductive amination was used to couple the APS to either soluble protein or PHB bead surface proteins^40^ (Fig. 3). The conjugation reaction yields were ranging from 4% to 36% (Supplementary Table 5).

Immunoassays using commercial anti-CPS (MenC) monoclonal antibodies (NIBS, UK) were performed to confirm the molecular identity of the CPS after the conjugation reactions. The CPS was only detected by the monoclonal antibody when chemically conjugated to protein-coated PHB beads or soluble proteins but not without conjugation (Supplementary Fig. 3).

**Figure 4:**
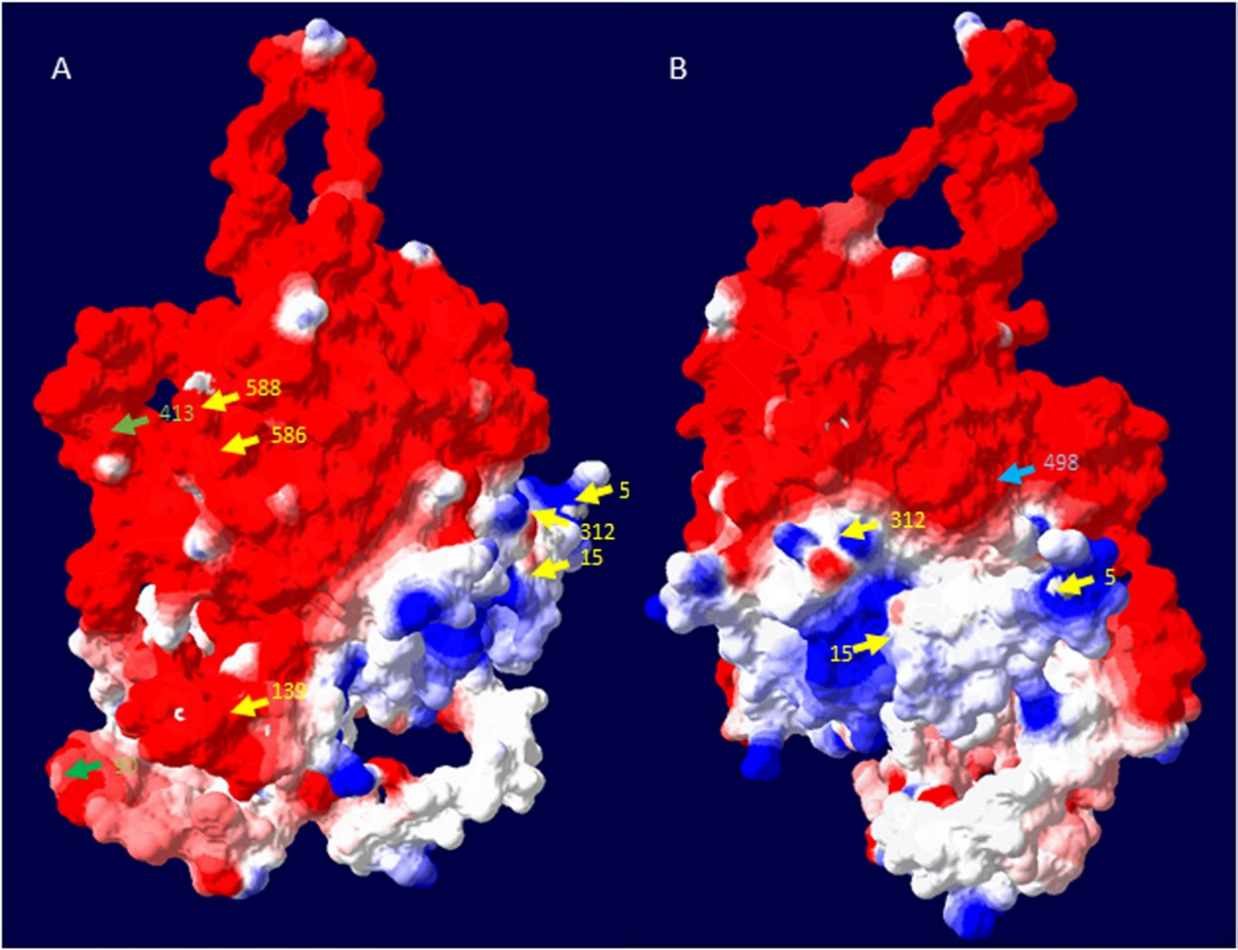
Structural models of PhaC depicting lysine residues proposed as sites conjugated to the activated polysaccharide. The structural model of PhaC was deduced from the crystal structure of the C-terminal region of the PHB synthase from *Ralstonia eutropha* (5HZ2) using i-Tasser server (https://zhanglab.ccmb.med.umich.edu/I-TASSER/). Resulting atom coordinates were displayed using the Swiss-PDB-Viewer (Version. 4.10) while computing the model surface using colouring based on the electrostatic potential. Arrows show the location of lysine residues and their sequence position, which were identified as possible sites for conjugation of the activated polysaccharide. Yellow and blue arrows show sites identified when PhaC was displayed on PHB beads, while blue arrows indicate sites which remained accessible for conjugation after PhaC was fused to NadA and displayed on PHB beads. The green arrows indicate conjugation sites accessible in PhaC only upon fusion to NadA. (**A**) Horizontal 90° clockwise rotation of the structural model shown in (**B**).

To locate conjugation sites in proteins coating the PHB beads, non-conjugated and conjugated PHB beads were subjected to trypsin/chymotrypsin digest and resulting peptides were analyzed by liquid chromatography-coupled tandem mass spectrometry (LC-MS/MS). The lower abundance of certain peptides upon conjugation, suggested a conjugation site (lysine residue) within the peptide **(**Supplementary Table 6**)**. Such identified lysine residues were depicted in the structural model of PhaC (Fig. 4). NadA fused to the N-terminus occluded 4 of the 7 sites identified without NadA. However, two new sites became accessible upon NadA fusion. Soluble NadA showed 24 conjugation sites of which 11 remained accessible after fusion to PhaC, i.e. display on PHB beads (Supplementary Table 6). NadA display on PHB beads generated 8 new accessible sites which were not detected in soluble NadA.

### Immunological properties of antigen-coated PHB beads displaying CPS

The immunological properties of CPS conjugated vaccine candidates were studied by immunizing mice (Table 2). All vaccinated animals remained healthy during the entire study. The conjugation of CPS to PHB beads had no impact on anatomical features when compared to the non-conjugated vaccine candidates as described above.

**Table 2:** Immunization groups for conjugated vaccine prototypes and features.

Immunization group	MenC amount (µg/mouse)	Protein amount (µg/mouse)	Adjuvant amount (mg/mouse)
MenC-NadA-PhaC	4	18	0.125
MenC-PhaC	4	14	0.125
MenC-NadA-His6	4	1.6	0.125
MenC-TD	4	1.5	0.125
NadA-PhaC	0	18	0.125
NadA-PhaC	0	18	0.125
PhaC	0	14	0.125
alum	0	0	0.125

**Figure 5:**
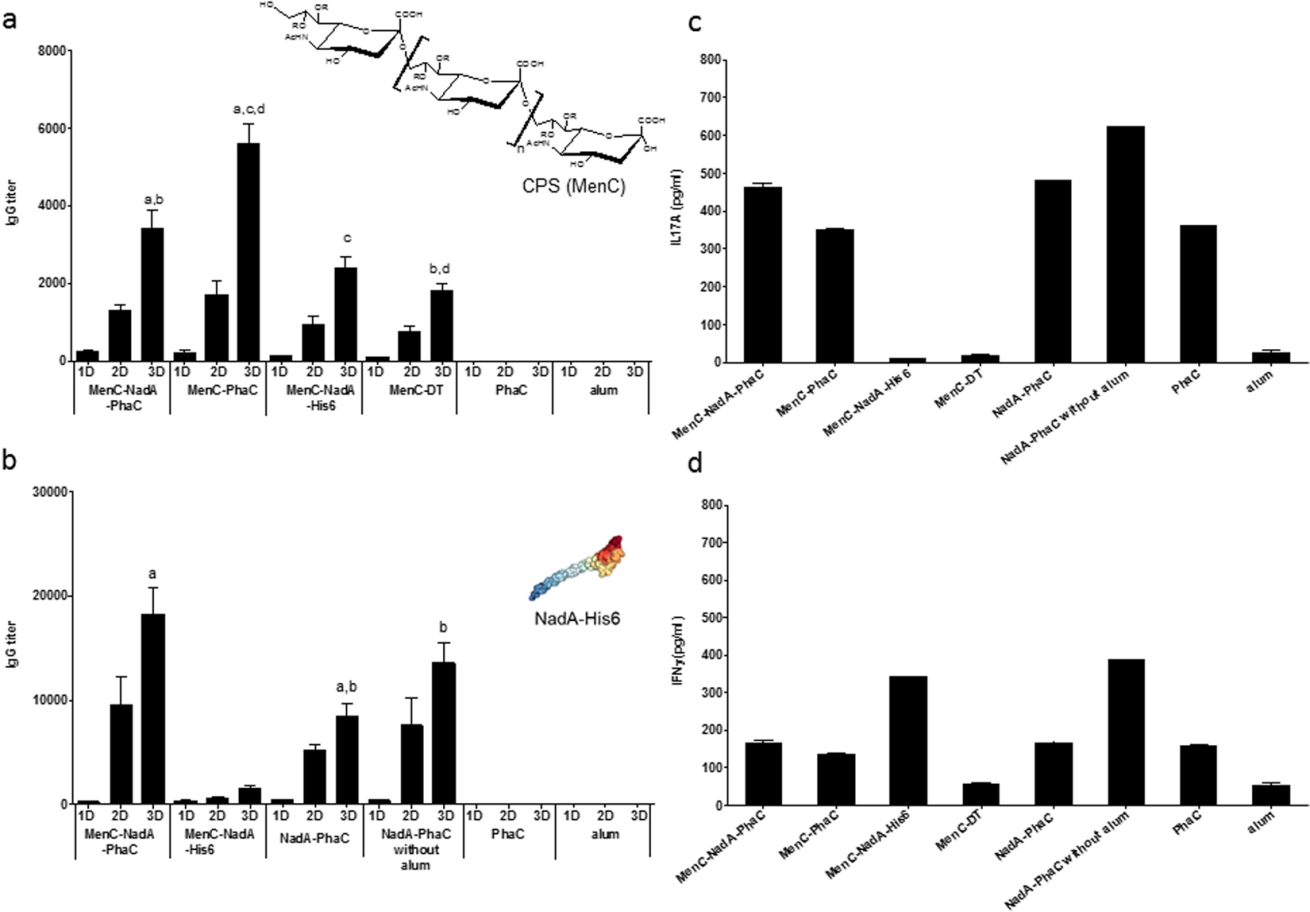
Immunogenicity studies of the various antigens conjugated to MenC. (**a**) Assessment of antibodies binding to Men C CPS; (**b**) Assessment of antibodies binding to NadA; (**c**) Assessment of IL17A cytokine levels; (**d**) Assessment of IFNɣ cytokine levels. In Fig. 5a and b, groups vaccinated with a formulation where the antigen under evaluation was not included were negative, (only control groups, PhaC and alum are shown). Cytokine production by spleen cell were evaluated by ELISA, under 10 µg/mL of NadA-His6 protein and 72 h of stimulation. Statistical differences between vaccination groups with *p* < *0.05* were labelled with letters. In Fig. 5a, IgG titers after 3D, from the group vaccinated with MenC-NadA-PhaC beads, was superior to the group vaccinated with MenC-DT (Mann-Whitney test, b (*p* < *0.01*)) while group vaccinated with MenC-PhaC beads was superior to the group vaccinated with MenC-NadA-PhaC beads, MenC-NadA-His6 and MenC-TD (ANOVA, Kruskal-Wallis test with Dunn’s multiple comparison post-test a,c,d (*p* < *0.01* and *p* < *0.001* respectively)). In Fig. 5b IgG titers after 3D from groups vaccinated with MenC-NadA-PhaC beads was superior to group vaccinated with NadA-PhaC beads (Mann-Whitney test a (*p* < *0.01*)) while group vaccinated with NadA-PhaC beads was inferior to the group vaccinated with NadA-PhaC beads without alum (Mann-Whitney test b (*p* < *0.05*)). Depicted is the variant 5 NadA, protein structure (4CJD) as derived from the protein data bank.

The humoral immune response was evaluated with the serum from each animal at different blood collection times, as mentioned in Materials and Methods. IgG titers against CPS increased after each vaccination dose for those vaccine candidates containing conjugated CPS (Fig. 5a). However, as was expected, animals from groups not exposed to CPS-containing vaccine formulations such as e.g. NadA-PhaC (data not shown) and non-antigen coated PHB beads (PhaC) and alum (placebo) were negative **(**Fig. 5a**)**. The strongest anti-CPS antibody induction was observed with non-antigen coated PHB beads conjugated to CPS (MenC-PhaC beads) (Fig. 5a).

The evaluation of the IgG titers against NadA showed an increasing trend with each vaccination dose in the groups that received NadA as part of the vaccine formulation (Fig. 5b). However, as was expected, animals from groups not exposed to NadA-containing vaccine formulations such as e.g. CPS conjugated to diphtheria toxoid (DT), non-antigen-coated PHB beads and alum (placebo) were negative (Fig. 5b). The highest IgG titers were elicited after the last vaccination doses for the positive groups. To investigate whether PHB beads show adjuvant properties, alum was omitted in one NadA-PhaC bead formulation which resulted in induction of an even statistically higher IgG titer compared to the respective formulation containing alum (Fig. 5b).

In order to study the Ig subclass profile against CPS and NadA, indirect ELISAs were performed using subclass-specific secondary antibodies (anti-IgG1, IgG2a, IgG2b, IgG3 and IgM). Serum from positive animals after the first and the third vaccination were assayed. Results, in the case of CPS, showed the predominant subclass elicited after vaccination was IgG1 reaching higher titers in the groups vaccinated with conjugated PHB beads than with the soluble proteins (Supplementary Fig. 4). The highest ratio of IgG/IgM was reached after the third dose, with the highest overall ratio found to be present in groups vaccinated with CPS conjugated to PHB beads (Supplementary Table 7). In the case of NadA, the IgG titer was the highest in mice immunized with NadA on PHB beads and predominantly composed of IgG1 subclass (Supplementary Fig. 5).

To study the possible mode (Th1, Th17) of immune response, splenocytes from 8 mice were pooled and re-stimulated with NadA-His6. The cytokines IL17A and INFγ were measured (Fig. 5c,d). Splenocytes from mice vaccinated with MenC-NadA-PhaC beads as well as NadA-PhaC beads produced significantly higher levels of IL17A than mice vaccinated with soluble MenC-NadA-His6, MenC-DT and alum only (Fig. 5c). The strongest stimulation of IFNγ production was found in splenocytes of mice vaccinated with MenC-NadA-His6 and with NadA-PhaC beads (no alum), respectively (Fig. 5d). The cytokine profile associated with antigen-coated beads suggested the induction of a mixed Th1/Th17 type immune response. However, more experiments will be required to further confirm this type of cellular immune response.

**Figure 6:**
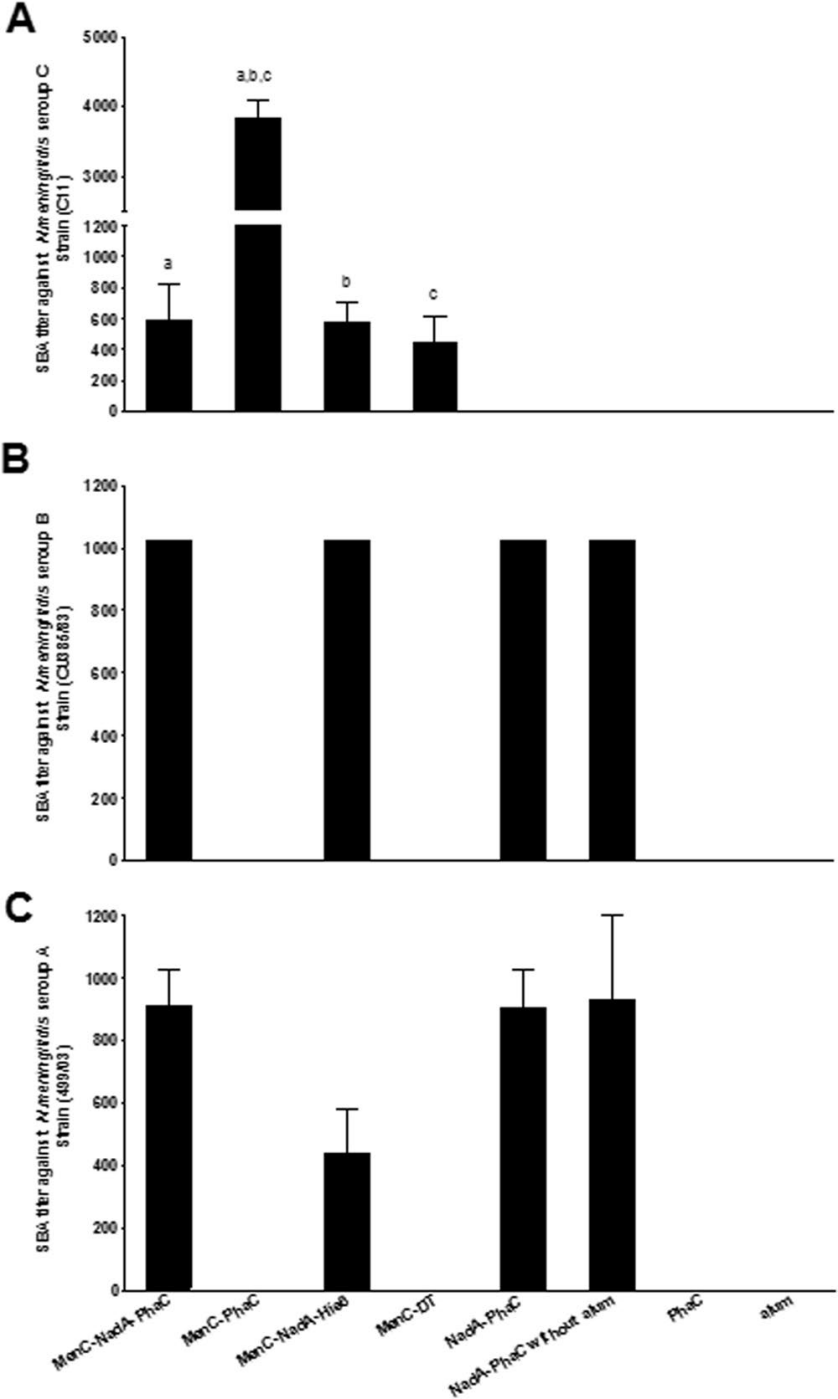
Bactericidal activity of various sera. (**A**) *N. meningitidis* serogroup C (Strain C11); (**B**) *N. meningitidis* serogroup B (Strain CU385/83); (**C**) *N. meningitidis* serogroup A (Strain 499/03). Bactericidal titers are expressed as mean ± SEM (8 mice each group) of the reciprocal value of the greatest sera dilution leading to ≥50% of killing. Statistical significant were determinate by an ANOVA by Kruskal-Wallis test with Dunn’s multiple comparison post-test in Fig. 6A and labelled with letters. Groups MenC-PhaC presented superior IgG levels than group MenC-NadA-PhaC, MenC-NadA-His6 and MenC-TD with a, b (*p* < 0.01) and c (*p* < 0.001).

The SBA is accepted as a correlate for assessing protective immunity against *N. meningitidis* infection^41,42^. Three *N. meningitidis* strains were used to investigate the functionality and possible cross-reactivity of the elicited antibodies. In the *N. meningitidis* serogroup C (C11 strain), only mice vaccinated with CPS-containing formulations presented specific and high SBA titers (Fig. 6A). Mice vaccinated with MenC-PhaC beads showed the highest SBA titer in comparison with the rest of the positive groups. However, in Fig. 6B the SBA titers against *N. meningitidis* serogroup B (CU385/03, Cuban strain) showed that mice, which received MenC-NadA-PhaC beads, NadA-PhaC beads, and soluble MenC-NadA-His6, respectively, presented high and similar SBA titers. In absence of NadA, the SBA was negative. In the case of *N. meningitidis* serogroup A (Mk499/03 strain), results showed that high SBA titers against this strain were achieved when NadA was present in the vaccine formulation (Fig. 6C). Nevertheless, mice vaccinated with MenC-NadA-PhaC beads induced a significantly higher SBA titer when compared to its soluble counterpart MenC-NadA-His6. No statistically significant differences were found between NadA-PhaC beads plus or minus alum which suggested that alum is not required to induce high SBA titer when NadA is displayed on PHB beads.

## Discussion

In this study, we systematically evaluated how *in vivo* assembled PHB inclusions could be refined as a possible immunogenic carrier for proteinaceous and carbohydrate-based antigens in the context of a bacterial extracellular pathogen such as *N. meningitidis*. As safe subunit vaccines composed of defined antigens are desirable, they often lack immunogenicity and broadly protective efficacy. Here we used bioengineering of *E. coli* to assemble neisserial protein antigen-displaying PHB beads and extended their antigen repertoire by chemical conjugation of a vaccine candidate CPS. Carbohydrate conjugate vaccines against *N. menigitidis* are the most common vaccines used world-wide. However, these vaccines do not prevent infection by serogroup B as their CPS, polysialic acid resembles a glycan present in human tissue and is hence not a suitable antigen^43,44^. Accordingly, alternative strategies based on the proteinaceous antigens, such as e.g. PorA and PorB (outer membrane proteins), were explored and resulted in the first vaccine generation against this serogroup^45,46^. Although serogroup independent, these vaccines faced coverage limitation due to an enormous variability of antigenic determinants in PorA and PorB^47^. New serogroup-independent protein-based vaccines were recently licensed against serogroup B (Bexsero®, GSK; Trumenba®, Pfizer). Soluble NadA and fHbp, due to their relevance in pathogenicity and their capacity to generate a strong bactericidal antibody response, were included in these licensed vaccines^16,19,48^. However, these antigens required the addition of adjuvants and their production is costly, prohibiting their wide use in developing and third world countries. Hence, we first explored the possibility of designing and producing a protein-based subunit vaccine requiring less antigen and implementing a cost-effective production process. Accordingly, a particulate antigen delivery system based on bacterial PHB inclusions was designed to directly enable the production of immunogenic antigens as a particulate vaccine. Particulate vaccines are inherently immunogenic as uptake by APCs is facilitated^49^. Bacterial PHB inclusions had previously been engineered to display *M. bovis* antigens and showed a strong cellular immune response resulting in protective immunity. The neisserial proteins, NadA^34^ and fHbp variant^50^, were each translationally fused to the PHB bead forming enzyme, PHB synthase (PhaC), and the respective fusion protein mediated assembly of PHB beads displaying the respective antigens when produced in an endotoxin-free mutant of *E. coli* (Fig. 1a and b). This endotoxin free *E. coli* mutant was used as a production strain to reduce the impact of endotoxin levels on the immune response^51,52^. A protein separation by SDS-PAGE confirmed the successful production of the full-length antigen-PhaC fusion and soluble proteins (Fig. 1c). The protein profile of the PHB beads showed some impurities, however, as had been previously shown, some *E.coli* proteins co-purify with PHB bead isolation but do not induce antibodies^31^. The chimeric antigen GNA2091-fHbp-G1 fused to the N*-*terminus of PhaC was more abundant on PHB beads when compared fHbp-G1-G1 fused to the C-terminus of PhaC (Fig. 1c), which suggested enhanced stability of the former fusion protein. Immunoblot and ELISA analysis using a monoclonal anti-fHbp antibody confirmed the presence of linear and conformational epitopes of fHbp in both GNA2091-fHbp-G1-PhaC and PhaC-fHbp-G1-G1 on PHB beads (Fig. 1d, Supplementary Fig. 1). Both PHB beads were used in animal trials.

The uptake of particulate vaccines by APCs and the respective antigen processing pathways were found to be dependent on physical properties such as size and surface charge^53^. The PHB beads produced and formulated in this study showed a mean particle size of less than 10 µm (Dx (50)). It remains unclear why PhaC beads and PhaC-fHbp-G1-G1 beads were produced at larger sizes (~500 nm) as observed by TEM when compared with the other PHB bead variants (~100 nm) (Fig. 1b). However, upon formulation in PBS or in alum, particle sizes increased for the majority of PHB bead variants (<10 µm) presumably due to electrostatic or hydrophobic interactions between the beads itself and/or alum (Supplementary Table 8).

Studies with polylactic acid (PLA) beads exhibiting a range of particle sizes (<2–8 µm, 10–70 µm and 50–150 µm) loaded with tetanus toxoid (TT) elicited IgG titers against TT dependent on the particle size. The optimum particle size range to elicit the highest IgG levels against TT was between 2–8 µm whereas the reduction of the particle size to less than 2 µm resulted in decreasing IgG titers. The lowest titers were achieved by those particles with the largest particle size 50–150 µm^54^. Fifis *et al*.^55^ found that carboxylated polystyrene spheres (20 nm-2 μm) conjugated with OVA elicited a strong humoral response while the highest IgG titers were obtained with particles showing a size of about 40 nm. Additionally, the uptake of antigen by immune system cells in the draining lymph node of the mice showed that 40 nm particles were preferentially taken up by mature/activated dendritic cells, while 1 μm particles were preferentially taken up by macrophages. This suggested that different APCs prefer different particle sizes for uptake^55^. The relationship between the surface charge and uptake by dendritic cells (DCs) and the immune response is controversial. Some authors proposed that particles with sizes >0.5 μm with positive surface charges enhanced association with DCs in comparison with particles showing negative surface charges^56^. However, other studies suggested that the surface charges are less critical for particles with a size of <0.5 μm in view of uptake efficiency by DCs^53^. The neisserial antigen-displaying PHB beads as well as the non-antigen-displaying control PhaC beads showed a negative surface charge at pH 7.5, which also corresponded with the theoretical isoelectric point of the PHB bead-associated PhaC or its fusion protein variant (Supplementary Table 3). How this negative surface charge contributes to uptake by APCs remains to be elucidated.

To develop an efficient broadly protective vaccine against extracellular pathogens like *N.meningitidis* the induction of a strong humoral immune response associated with the bactericidal activity is desirable and correlated with protective immunity^57^. For the meningococcal conjugate vaccine the protection correlate is 2 µg/mL of the IgG antibodies with an SBA ≥4 when human complement is used as a complement source^58,59^. Although the same assays and similar criteria were followed for serogroup B, a correlation between SBA activity and protective immunity is not clearly defined, which further impairs the development of a vaccine against this serogroup. Immunological properties of the various PHB beads were assessed in mice (Fig. 2a) and revealed that the particulate NadA-PhaC beads induced a significantly greater humoral immune response against NadA than its soluble counterpart NadA-His6 with p < 0.01 (Fig. 2b). In contrast, GNA2091-fHbp-G1-PhaC beads or fHbp-G1-G1-PhaC beads induced high and specific IgG levels but less than obtained with the soluble GNA2091-fHbp-G1-His6 (Fig. 2d). Here the display of cell surface antigens on the PHB bead surface was intended to simulate the natural surface exposition of the antigen towards induction of an immune response associated with the production of specific functional antibodies^60^. However, the particulate surface display is only one parameter while many factors, such as e.g. antigen structure, size or charge of the particles, can influence the immune response.

The correlation between Ig subclasses and bactericidal activity in the context of meningococcal diseases was found to differ between humans and mice based on pre-clinical and clinical trials^61–64^. In humans it is known that IgG1 and IgG3 are the best IgG subclasses contributing to efficient bactericidal activity^62,64^. However, analysis of mouse monoclonal antibodies specific for epitope P1.16 of meningococcal PorA had shown a hierarchy of IgG3» IgG2a/b» IgG1 with respect to bactericidal activity^63^. In our study, IgG1 was the predominant subclass after immunization with PHB beads (Fig. 2c,e), while IgG2b induction was enhanced when antigens were displayed on PHB beads. Similar results were presented by Parlane *et al*.^65^, when antigens from *M. bovis* were displayed on PHB beads, suggesting that antigen-displaying PHB beads promote a mixed Th1/Th2 immune response. The immunization with NadA-PhaC beads elicited not only high IgG1 and IgG2b levels but in addition induced antibodies mediating killing of heterologous *N. meningitidis* serogroup A (strain MK499/03, NadA, Allele 3)^38,66^ through classical complement activation pathways (Fig. 2f). Less SBA was observed in mice immunized with soluble NadA-His6. Since NadA-PhaC beads showed superior immunological properties, when compared with its soluble counterpart and the other protein antigen displaying PHB beads, they were selected for conjugation experiments adding a carbohydrate-based antigen.

To further broaden protective efficacy, bioengineered antigen-displaying PHB beads (NadA-PhaC beads and PhaC beads) were subjected to chemical conjugation of carbohydrate-based antigens (Fig. 3). The CPS (MenC) of serogroup C had been included in all of the licensed conjugate vaccines because of its epidemiological relevance. Hence it was selected and conjugated to various PHB beads, soluble NadA-His6 or DT (controls), respectively (Fig. 3). To estimate the conjugation efficiency, the CPS/protein ratio was determined by colorimetric assays and results showed low percentages of CPS in the final conjugates, as was previously described^67,68^. The molecular identity of the CPS after conjugation was confirmed by ELISA (Supplementary Fig. 3).

Assessment of conjugations sites suggested that PhaC shows an extended accessible surface area while being attached to PHB beads and that these sites might be occluded by N-terminal fusion partners (Supplementary Table 6, Fig. 4).

Two of the sites, K90 and K139, were also identified by Hooks and Rehm^69^ who used biotinylation to identify surface exposed residues. In their study, further residues (K77, C382, C459, and K518) were found to be accessible which was presumably due to the smaller size of biotin. As fusion of NadA to PhaC still enabled access to 11 of the 24 conjugation sites found in soluble NadA and enabled access to further 8 lysines, it is suggested that NadA is extensively exposed at the surface of the PHB beads^69^.

NadA-PhaC beads were chosen as the protein antigen displaying beads for conjugation because of their superior immunological properties when compared with its soluble counterpart and the other protein antigen-displaying PHB beads (Fig. 2 b,c and f).

The imminent importance of production of antibodies and their functionality (SBA) have been extensively used to evaluate the efficacy of the meningococcal conjugate and protein vaccines^41,60,68^. In our study, all CPS conjugate vaccine candidates induced considerable IgG titers against MenC, when compared with the negative controls (Fig. 5a). As was expected, the IgG levels increased after each dose, achieving the highest values after the third dose. These results are an indication of the improvement in the immune response against the CPS once they are conjugated to an immunogenic carrier^70^. However, the group vaccinated with MenC-NadA-PhaC beads was statistically inferior to the MenC-PhaC bead group and superior to MenC-DT group (conjugate control) with *p* < 0.001. The better performance of MenC-PhaC beads could be due to the greater CPS loading per mg protein compared to MenC-NadA-PhaC beads (Supplementary Table 5), which would also justify further optimizing conjugation efficiency. The predominant subclass induced against MenC CPS was IgG1 followed by discrete levels of IgG2a and IgG2b, in the group MenC-NadA-PhaC beads (Supplementary Figure 4). This result indicated a possible mixed Th1/Th2 type immune response.

Additionally, NadA-bearing vaccine candidates co-displaying conjugated CPS also induced significant levels of anti-NadA antibodies. The IgG levels presented by the MenC-NadA-PhaC group were superior to the group immunized with NadA-PhaC+ alum with *p* < 0.001, (Fig. 5b). The predominant Ig subclass against NadA in groups vaccinated with PHB beads was IgG1, however in the absence of CPS, discrete levels of IgG2a, b were observed (Supplementary Fig. 5). In addition to this possible Th2-type immune response, preliminary cytokine analysis suggested that the MenC-NadA-PhaC and NadA-PhaC beads also induced a cell-mediated immune response, presumably of mixed Th1/Th17 pattern, while soluble antigens induced Th1 but not Th17 responses (Fig. 5c,d). Interestingly, omission of alum in the NadA-PhaC beads formulation increased immunogenicity toward Th1-, Th2-, Th17-type immune responses, which suggested self-adjuvanting properties of the PHB bead carrier system (Fig. 5b,c,d). More experiments should be performed to elucidate the underlying immunological mechanism.

The SBA titers against the three serogroups A, B, C confirmed the superior performance of NadA and CPS co-displayed on PHB beads when compared to their soluble counterparts (Fig. 6). While NadA on PHB beads mediated protective immunity against serogroups A and B, the co-display of MenC expanded coverage to serogroup C (Fig. 6). This suggested that combining protein and carbohydrate antigens on the surface of particulate carriers provides design space towards the development of broadly protective vaccines.

## Conclusion

Overall, this study showed that PHB beads displaying selected neisserial antigens can be recombinantly produced in an endotoxin-free *E. coli* mutant and that these PHB beads can be further modified by chemical conjugation to co-display carbohydrate antigens. Antigen-displaying PHB beads were immunogenic, mediating strong and specific humoral and cell-mediated immune responses leading to protective immunity. Hence the PHB bead-based particulate vaccine approach holds the promise to be applicable not only for protection against intracellular pathogens but also extracellular pathogens such as *N. meningitidis*.

## Materials and Methods

### Ethics statement

The experiments described in this study were approved by The Institutional Ethics Committee for the Care and Use of Laboratory Animals of Finlay Vaccine Institute, Havana, Cuba. The guidelines and regulations of this committee were written following the National Regulations about Principles of Good Practices of Non-Clinical Laboratory Sanitary and Environmental Safety (No. 39/04) and guidelines provided by the Canadian Council on Animal Care^71^. The protocols were approved by this ethics committee. All methods were performed in accordance with the relevant guidelines and regulations. All efforts were made to minimize animal suffering and to reduce the number of animals used.

### Construction of plasmids mediating production of PHB beads

Genes encoding NadA, GNA2091-fHbp-G1 and fHbp-G1-G1^16,34,50,72^ were synthesised by Genscript Corporation (USA) employing codon optimization for *E. coli*^14,50,72,73^. The amino acid sequence of the NadA was reconstituted as a combination of the whole sequence allele 1 (24–351 aa) (Genbank AF452481), three repeats of the immunodominant peptide (52–70 aa), two repeats of the anti-adhesion peptides (25–30aa) and a selected region (94–11aa) from the same allele 1^17,72^. In the case of GNA2091-fHbp-G1 and fHbp-G1-G1 proteins, the amino acid sequence was composed as mentioned elsewhere^14,17,50^.

Hybrid genes encoding fusion proteins comprised of antigen and PhaC were generated as previously described^25^. DNA sequencing confirmed construction of plasmids pET-14b-nadA-phaC, pET-14b- gna2091-fhbp-g1-phaC and pET-14b- phaC-fhbp-g1-g1 (Supplementary Table 1).

### Construction of plasmids for production of soluble His-tagged proteins

Genes encoding NadA and GNA2091-fHbp-G1 were amplified from pUC57-nadA and pUC57-gna2091-fhbp-g1 by PCR using primers listed in (Supplementary Table 1). Six histidine residues were introduced at the C-terminus of each protein using PCR and respective oligonucleotides^50,74^. Respective coding regions were cloned into pET-14b_NanA_PhaC replacing NanA_PhaC after hydrolysis with *Nde*I/*Bam*HI restriction enzymes. The DNA sequences of new plasmids pET14b-nadA-his6 and pET14b-gna2091-fhbp-g1-his6 were confirmed by DNA sequencing.

### Production, isolation and purification of PHB beads and soluble proteins

*ClearColi*^TM^^ 51^ harbouring pMCS69 was transformed with plasmids mediating PHB bead formation. Cells were cultivated (48 h, 25 °C at 200 rpm), harvested and subjected to mechanical cell disruption. Bead isolation and purification procedures were previously described^52,75^. In the case of soluble proteins, *ClearColi*^*TM*^ harboring pET14b-nadA-his6 and pET14b-gna2091-fhbp-g1-his6 were cultivated (24 h, 25 °C at 200 rpm) and lysed by sonication. Proteins were purified using a Ni-NTA Fast Start Kit column (Qiagen, Germany)^76^.

### Conjugation of MenC polysaccharide (CPS) to carrier proteins

One mg of protein (PBS, pH 7.0) was conjugated to 5 mg (APS) from *Neisseria meningitidis* serogroup C by reductive amination. NaBH_3_CN was added as a reducing agent (1/5 of the amount of carbohydrate). The reaction was stirred for 72 h at 37 °C^77^. Reaction products were purified by successive washes with NaCl solution 0.9% (w/v) until the absorbance in resorcinol assay (colorimetric assay for determination of sialic acid) was equal to the blank^70^.

### Transmission electron microscopy analysis (TEM)

*ClearColi*^*TM*^ harboring plasmid pET-14b-nadA-phaC, pET-14b-gna2091-fhbp-g1-phaC, pET-14b-phaC-fHbp-g1-g1 and pET-14b-phaC, as well as the respective purified PHB beads were was analyzed by TEM. Samples were prepared as described previously^78^.

### Measurement of PHB bead size distribution and surface charge

Size distribution of the particles and the zeta potential were measured using the Mastersizer 3000 particle sizer (Malvern instrument, United Kingdon) and the Zetasizer Nano ZS (Malvern instrument, United Kingdom), respectively. Samples were prepared as 0.1% (w/v) of the wet PHB beads in saline solution. The pH values were adjusted with HCl.

### NMR spectroscopy

^1^H NMR spectra in deuterium oxide were recorded on a Bruker Avance 500 MHz spectrometer at 313 K using standard parameters. Spectra were referenced to the residual HOD peak at 4.50 ppm. The results were interpreted as described elsewhere^39^.

### Protein analysis

NadA-PhaC, GNA2091-fHbp-G1-PhaC, fHbp-G1-G1-PhaC fusion proteins and PhaC wild type on PHB bead as well as NadA-His6 and GNA2091-fHbp-G1-His6 were analyzed by SDS-PAGE^79^. Immunoblotting was conducted as previously described^69^. Anti-Meningococcal Factor H binding protein variant 1 (JAR 4) monoclonal antibody from NIBSC was used to characterise the molecular identity of fHbp in the fusion and soluble version of this protein. A monospecific polyclonal antibody was used to confirm the identity of the PhaC. All images were obtained using the GEL-DOC 2000 (Bio-Rad Laboratories, USA) and analysed using Image Lab Software (Version 3.0 build 11, Bio-Rad Laboratories, USA). Proteins were further identified using tryptic peptide analysis by matrix-assisted laser desorption ionization time-of-flight/mass spectrometry (MALDI-TOF/MS). For conjugation site analysis tryptic and chymotryptic peptides were analyzed by liquid chromatography-coupled LTQ-Orbitrap tandem mass spectrometry (LC-MS/MS).

### Protein and carbohydrate quantification

Ratio ng of the fusion protein/ mg of the wet bead was determined as mentioned above^80^. The protein concentrations of NadA-His6 and GNA2091-fHbp-G1-His6 were determined by BCA colorimetric assay (Pierce™ BCA Protein Assay Kit)^81^. Ratio mg of polysaccharide / mg of protein in the conjugate was determined by Lowry and the resorcinol colorimetric assay^82,83^.

### Immunization schedule for proteinaceous antigens

Eight immunization groups comprising each 6 animals of 5–6 weeks old Balb/c mice were used in this study. Mice were purchased from CENPALAB (Centro Nacional para la Producción de Animales de Laboratorio, La Habana, Cuba). Groups were organized as shown in Table 1.

All immunogens were mixed with alum (ALHYDROGEL, Brenntag Biosector, Denmark) at 4 °C for 18 h^84^. The inoculation route was subcutaneous. The immunization occurred at 0, 21 and 35 days^50^ and blood collection at days 0, 7 (1D), 28 (2D) and 42 (3D) (Fig. 2a)^84^. Blood samples here were collected from the retro-orbital plexus.

### Immunization schedule for conjugated vaccine prototypes

Eight immunization groups were prepared including 8 animals each, from the same breed as previously mentioned. Groups were organized as shown in Table 2.

Except in group NadA-PhaC without alum, all vaccines were mixed with alum at 4 °C for 18h. Immunizations occurred at 0, 14 and 28 days and the blood samples were collected at days −4, 14 (1D), 28 (2D) and 35 (3D) (Fig. 3)^70^.

### Assessment of anti-NadA and anti-fHbp antibody titers in mice

Anti-NadA and anti-GNA2091-fhbp-G1 IgG levels were measured using an indirect ELISA. Maxisorp 96-well plates (NUNC) were coated using NadA-His6 or GNA2091-fHbp-G1-His6 followed by incubation overnight at 4 °C.The rest of the ELISA was performed as mentioned above^80^. IgG titers were calculated as the reciprocal antibody titers, representing the Dilution Factor (DF) required to obtain half of the maximum level of absorbance, per animal. The results are expressed as the (mean ± SEM) of 6 or 8 animals.

### Assessment of anti-MenC antibody titers in mice

Anti-MenC CPS IgG antibodies levels were measured using a standardized indirect ELISA with some modification^85^. A brief explanation, Maxisorp 96-well plates (NUNC) were coated using a mix between Men C Capsular polysaccharide and methylated human albumin (HSA) with a final concentration of 5 µg/ml each, followed by incubation overnight at 4 °C. The rest of ELISA was performed as mentioned above. The results are expressed as the (mean ± SEM) of 8 animals.

### Analysis of the Ig subclass profile in sera

The Ig subclass profile against NadA, fHbp and Men C CPS was analyzed by using an indirect ELISA as described above. Plates were coated as previously described. To detect the various Ig subclass, plates were incubated with the respective anti-IgG1, IgG2a, IgG2b, IgG3 and anti-IgM antibodies (derived from goat) (Sigma-Aldrich, St. Louis, MO) using a DF of 1: 2500. Plates were developed using the anti-goat IgG peroxidase conjugate and the respective substrate as described above. The results are expressed as (mean ± SD) of two replicas per subclass (see immunization schedule for proteinaceous antigens) or as (mean ± SEM) of 8 animals per subclass (see immunization schedule for conjugated vaccines).

### Analysis of the cytokine production

Spleens were removed from each mouse 7 days after the last vaccination dose (see immunization schedule for conjugated vaccines), and cells were recovered by perfusion and pooled. Erythrocytes were then removed by osmotic shock, using NaCl 0.2%. The cells were counted, and the viability was tested by trypan blue exclusion staining. Spleen cells were adjusted to 4 × 10^6^ cells/mL and were cultured in DMEM medium supplemented with 50 μg/mL of gentamycin, 2 mmol/L of L-glutamine, 1 mmol/L of sodium pyruvate, 15 mmol/L of HEPES and 10% of inactivated FCS (all from Sigma-Aldrich). Isolated spleen cells were stimulated *in vitro* with NadA-His6 (10 µg/mL) or Concanavalin A (positive control) for 72 h. The level of IFNγ, and IL17A in the supernatant of the culture was measured by double sandwich capture ELISA using commercial monoclonal antibodies following manufacturer instructions (Mabtech, Sweden)^86^. Results are expressed in (mean ± SD) of two replicas per immunization group.

### Serum bactericidal assay

The bactericidal assay was performed following the protocol described by Borrow *et al*.^37^. The targeted strain was *N. meningitidis* serogroup A (Strain Mk499/03)^38,66^, serogroup B (Strain CU385/83)^11^ and serogroup C (Strain C11). Baby rabbit complement source was used to perform the assay^59^. SBA titers were expressed as mean ± SEM per group of the reciprocal value of the greatest sera dilution leading ≥50% of bacteria killing.

### Statistical analyses

GraphPad Prism 5.00 (San Diego, USA) software was used for statistical analysis of data. Statistical differences between all groups were calculated by one-way analysis of variance (ANOVA) with Kruskal–Wallis non-parametric test. When significant differences were found, the Dunn’s post-test was used considering significance at *p* < 0.05. When the comparison was just between two groups the Mann-Whitney test was performed and statistical differences were considered significant at (*p* < 0.05).

### Data analysis

All relevant data are available from the authors upon request.

## Electronic supplementary material

Supplementary Material
