# Supplementary material for: Bioengineered polyester beads co-displaying protein and carbohydrate-based antigens induce protective immunity against bacterial infection
Source: Sci Rep. 2018 Jan 30;8:1888. doi: 10.1038/s41598-018-20205-7 (PMC5789850; doi:10.1038/s41598-018-20205-7)
Supplement: Supplementary file 1 — Supplementary Material [file 41598_2018_20205_MOESM1_ESM.pdf]

Supplementary material

Bioengineered polyester beads co-displaying protein and carbohydrate-based antigens induce protective immunity against bacterial infection

Majela González-Miro[1,2], Laura M Rodríguez-Noda[2], Mildrey Fariñas-Medina[2], Barbara Cedré-Marrero[2], Sandra Madariaga-Zarza[2], Caridad Zayas-Vignier[2], Mabel Hernández-Cedeño[2], Torsten Kleffmann[3], Dagmar García-Rivera[2], Vicente Vérez-Bencomo[2],

Bernd H. A. Rehm[1, 4*]

[1]Institute of Fundamental Sciences, Massey University, Palmerston North, New Zealand

[2]Finlay Vaccine Institute, La Havana, Cuba;; [3]Biochemistry Department, University of Otago, New Zealand; [4]Centre for Cell Factories and Biopolymers, Griffith Institute for Drug Discovery, Griffith University, Nathan, Queensland, Australia

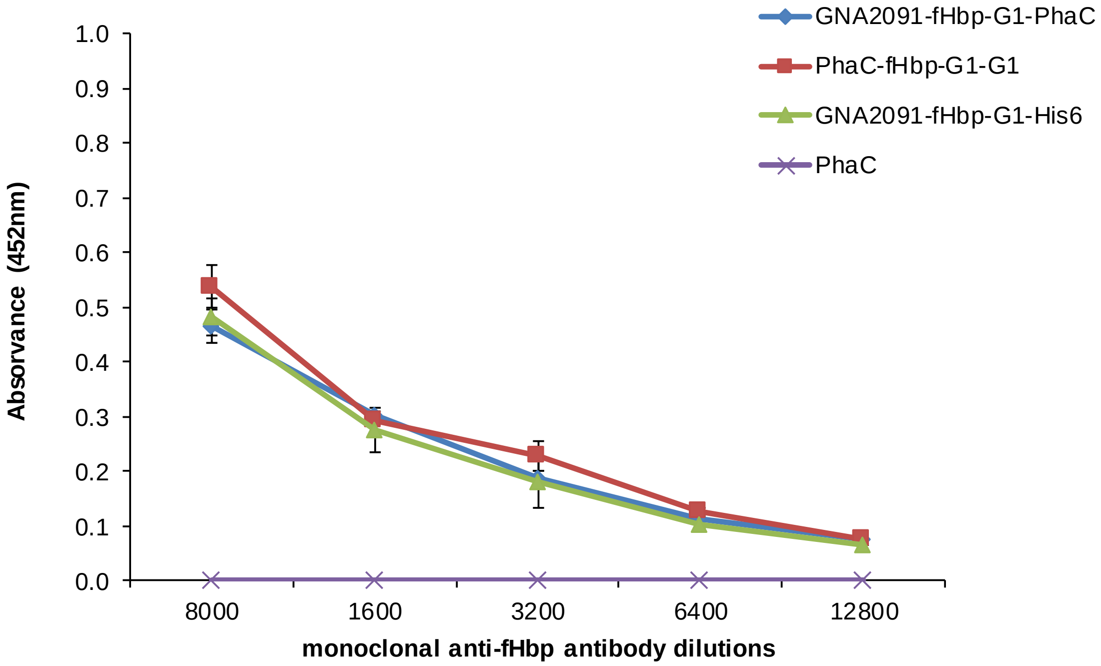

Supplementary Figure 1:

FHbp confirmation of molecular identity and bead display by ELISA using a commercial monoclonal anti-fHbp antibody (JAR4, NIBCS, UK).

Maxisorp 96-well plates (NUNC) were coated using 0.5 µg/ml of each protein and the rest of assay was performed as mentioned above[1]. The results are expressed as the mean ± SD of two replicas per sample.

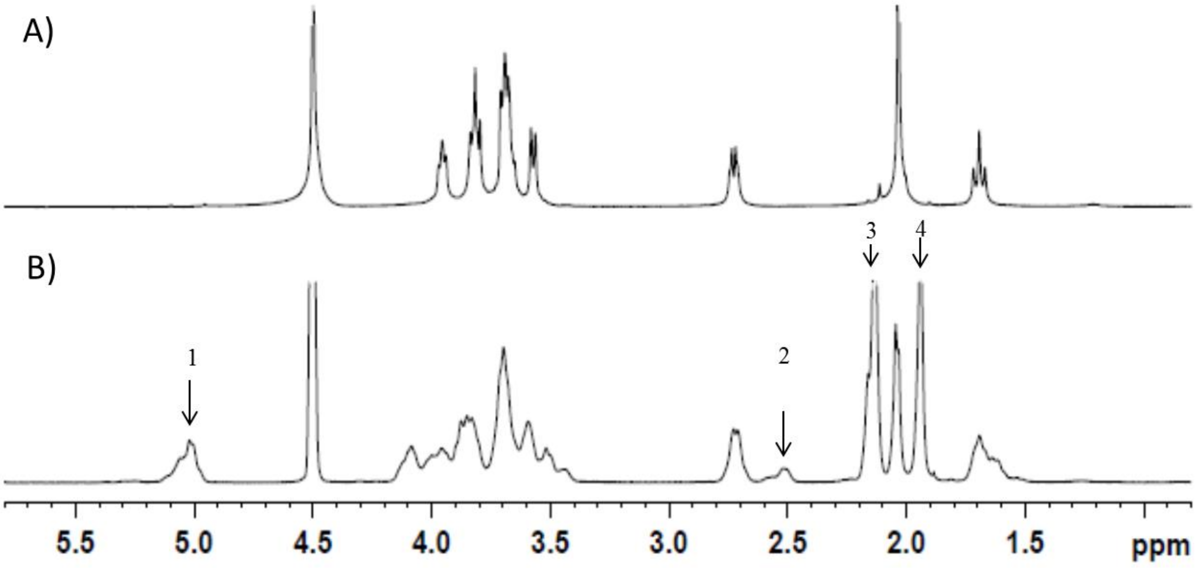

Supplementary Figure 2:

^{1}H NMR monodimensional spectra of CPS.

A) Activated polysaccharide (APS) and B) Capsular polysaccharide (CPS), both from *Neisseria meningitidis* Serogroup C. The peaks 1,2,3, and 4 are missing in the Aps spectrum because the O-Acetyl groups were removed during activation reaction, but the rest of the chemical structure are conserved after activation as mentioned above [2].

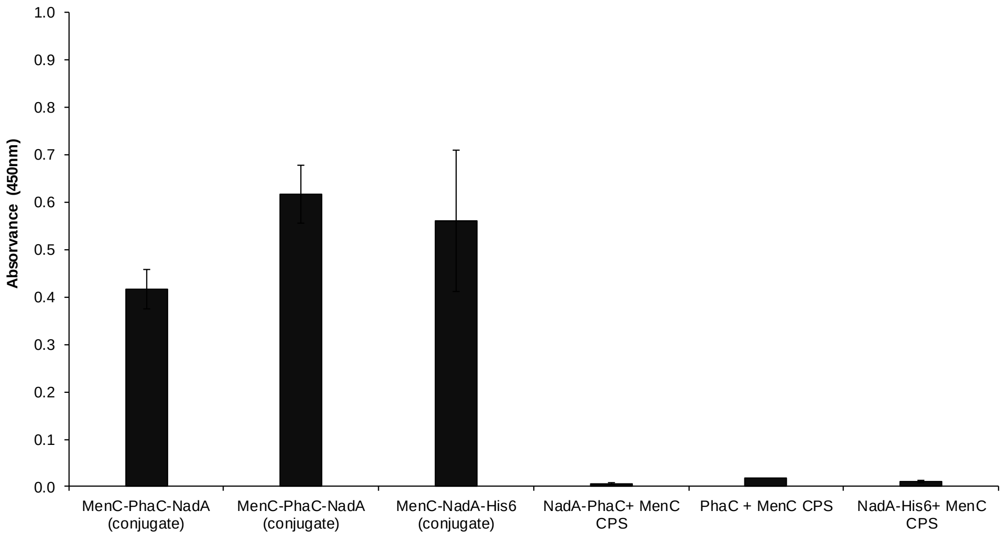

Supplementary Figure 3:

MenC confirmation of molecular identity by ELISA using a commercial anti-CPS (MenC) monoclonal antibody (NIBS, UK).

Maxisorp 96-well plates (NUNC) were coated using 5 µg/ml of MenC CPS conjugated or mixed to NadA-PhaC beads, PhaC wild type beads and NadA-His6 the rest of assay was performanced as mentioned above[1]. The results are presented as the mean ± SD of two replicas. The results are expressed as the mean ± SD of two replicas per sample.

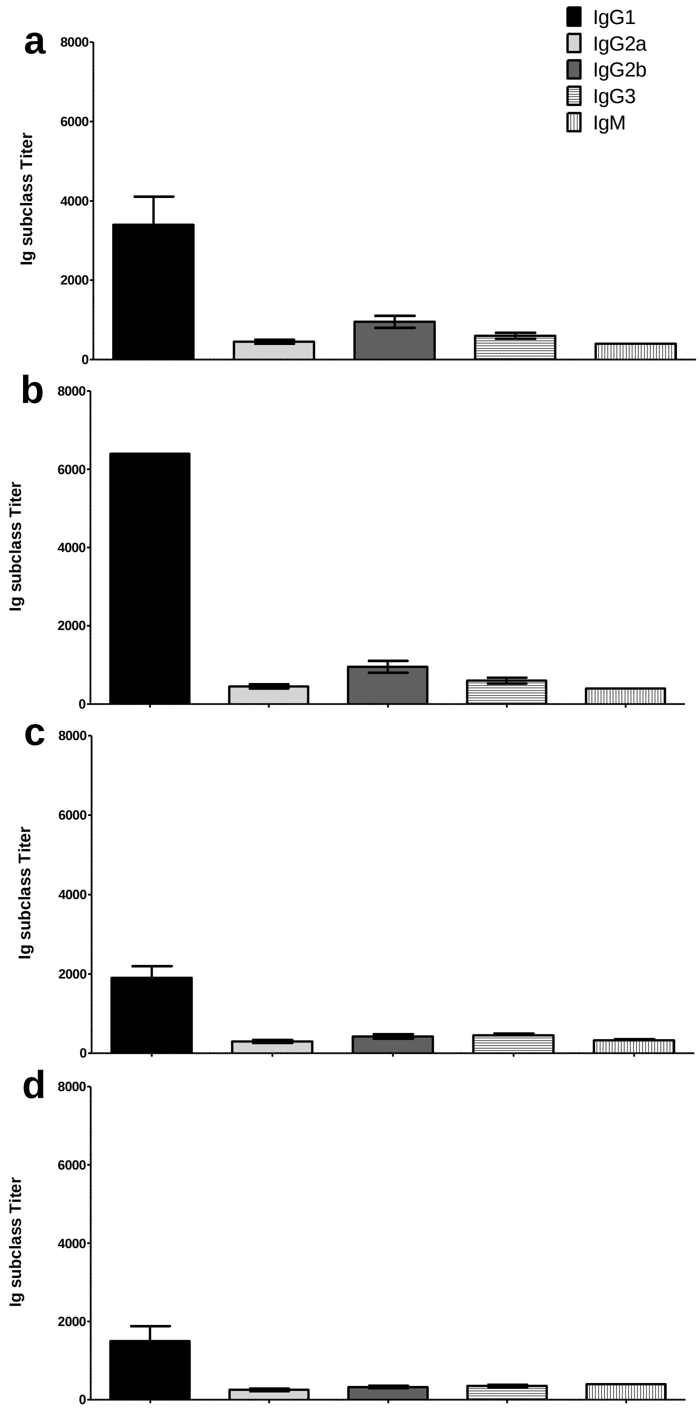

Supplementary Figure 4:

Assessment of IgG subclass binding to MenC evaluated by ELISA.

Results are expressed as the mean ± SEM of 8 animals and the titer was calculate as mentioned above. **a.** animals vaccinated with 4µg of MenC conjugated to NadA-PhaC beads; **b.** animals vaccinated with 4µg of MenC conjugated to PhaC wild type beads; **c.** animals vaccinated with 4µg of MenC conjugated to soluble NadA-His6; **d.** animals vaccinated with 4µg of MenC conjugated to DT.

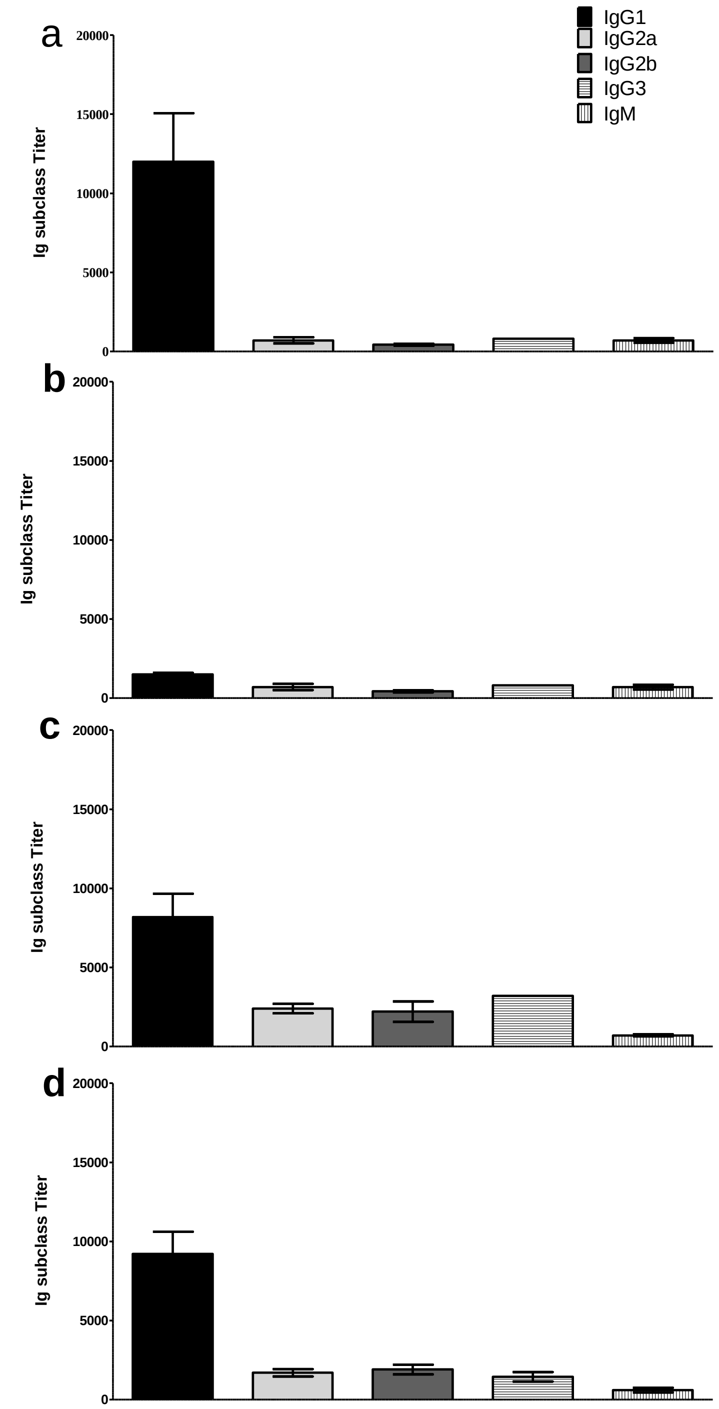

Supplementary Figure 5:

Assessment of IgG subclass binding to NadA protein evaluated by ELISA.

Results are expressed as the mean ± SEM of 8 animals and the titer was calculate as mentioned above. **a.** animals vaccinated with 4µg of MenC conjugated to NadA-PhaC beads; **b.** animals vaccinated with 4µg of MenC conjugated to soluble NadA-His6; **c.** animals vaccinated with 18µg of protein from NadA-PhaC beads; **d**. animals vaccinated with 18µg of protein from NadA-PhaC beads without alum.

Supplementary Table 1: Strains, Plasmids and primers

Strains, plasmid and primers	Features	Source
	**Strains**	
XL1-Blue	*recA1 endA1 gyrA96 thi-1 hsdR17 supE44 relA1 lac* [*F´ proAB lacI^{q}ZΔM15 Tn10* (Tet^r)]	Novagen
Clear Coli	*F– ompT hsdSB (rB - mB -) gal dcm lon λ (DE3 [lacI lacUV5-T7 gene 1 ind1 sam7 nin5]) msbA148 ΔgutQ ΔkdsD ΔlpxL ΔlpxM ΔpagP ΔlpxP ΔeptA (*LPS negative)	[3]
	**Plasmids**	
pET-14b	*Ap^R* and T7 promoter	Novagen
pET-14b-phaC	pET-14b version, holding *phaC* gene fragment	[4]
pUC57-nadA	pUC57 version, ColE1 origin, *Ap^R* holding *Nde*I /*nadA* gene/*Spe*I.	GenScript
pUC57-gna2091-fhbp-g1	pUC57 version, ColE1 origin, *Ap^R* holding *Nde*I */gna2091-fhbp-g1* gene/*Spe*I.	GenScript
pUC57-fhbp-g1-g1	pUC57 version, ColE1 origin, *Ap^R* holding *XhoI/ fhbp-g1-g1*gene/ *BamH*I	GenScript
pMCS69	CmR; T7 promoter, pBBR1MCS derivative containing *phaA* and *phaB* genes from *Ralstonia eutropha* co-downstream to lac promoter	[5]
pET14b_NanA_PhaC (reversed)	Ampicillin resistance and T7 promotor, holding *nana* gene fused translationally to 3' end of phaC gene	[6]
pET-14b-nadA-phaC	pET-14b-phaC version, holding *nadA* gene fused translationally to 3' end of *phaC* gene	This study
pET-14b-gna2091-fhbp-phaC	pET-14b-phaC version, holding *gna2091-fhbp-g1* gene fused translationally to 3' end of *phaC* gene	This study
pET-14b-phaC-fhbp-g1-g1	pET-14b-phaC version, holding *fhbp-g1-g1* gene fused translationally to 5' end of *phaC* gene	This study
pET14b-nadA-hist6	Ampicillin resistance and T7 promotor, holding a *Nde*I/ *nadA hist-tag* /*BamH*I gene.	This study
pET14b-gna2091-fhbp-g1-hist6	Ampicillin resistance and T7 promotor, holding *Nde*I/ *gna2091-fhbp-g1-his-tag*/*BamH*I gene.	This study
	**Primers**	
nadA fwr (*Nde*I, underline restriction site)	5'AAA<u>CATATG</u>GCTACCTCAGATGATGATGTCAAAAAGGCGG3'	This study
nadA rev (*BamH*I, underline restriction site)	5'AAA<u>GGATCC</u>TCAGTGGTGGTGGTGGTGGTGTGCTTTCACTTTTGCGTCCACATTTTGTTTGTTTTCATTCAC3'	This study
gna2091-fhbp-g1 fwr (*Nde*I, underline restriction site)	5'AAA<u>CATATG</u>TGCGTTTCGGCGGTTATTGGCTCAGCGGCGGTTG3'	This study
gna2091-fhbp-g1 rev (*BamH*I, underline restriction site)	5'AAA<u>GGATCC</u>TCAGTGGTGGTGGTGGTGGTGCTGTTTTGCCGCCAGACCAATGTGACGAATACCGTTGACC 3'	This study

Supplementary Table 2: Identification of fusion proteins by peptide fingerprinting analysis (MALDI-TOF/MS)

**Fusion proteins**	**Peptides fragment identified by MALDI-TOF/MS.***
NadA-PhaC	A11-K47, L134-K146, Q267-R309, A317-K341, V482-R498, I535-K552, F608-641, I657-K691, Y712-K874, L928-K979, R1025-R1035
GNA2091-fHbp-G1-PhaC	T31-R45, G52-R64, Q78-105, V170-R180, D212-R224, F259-R263, I315-R347, S388-R395, G405-R414, V641-R476, I503-R514, A531-R565, F586-R620, I635-R647, Y690-R745, E811-R826, H873-K938, F940-K958, A1004-R1023
PhaC-fHbp-G1-G1	A2-W31, A59-Y65, L161-L171, T191-T218, T232-L240, V262-F273, I305-L316, A333-L346, R386-W404, L546-W554, R575-Y584, A592-I638, T643-Y665, T725-M731, R738-Y77, K793-Y822

* Red bold, identified peptides belonging to the respective neisserial antigen.

Supplementary Table 3:

Correlation between Zeta potential and pH of various PHB beads.

**PHB beads**	**Theoretical pI of the respective protein***	**Zeta potential (mV) pH (3.5) ****	**Zeta potential (mV) pH (5.5) ****	**Zeta potential (mV) pH (7.5) ****
NadA-PhaC on PHB beads	5.01	5.20 ± 0.9	-13.3 ± 2.2	-20.4 ± 3.2
GNA2091-fHbp-G1-PhaC on PHB beads	6.77	16.2 ± 1.4	-8.9 ± 0.9	-16.5 ± 2.5
PhaC-fHbp-G1-G1 on PHB beads	6.21	11.1 ± 2	-8.4 ± 0.7	-17.9 ± 2
PhaC, (non-antigen displaying PHB beads)	6.08	14.4 ± 1.8	-5.7± 0.9	-16.8± 0.35

* ExPASy - Compute pI/Mw tool (theoretical isoelectric point (pI)); ** mean of 3 replicates ± SD.

Supplementary Table 4:

Amount of neisserial antigen attached to PHB beads and immunization doses.

**PHB beads**	**neisserial antigen/ wet Bead (µg/mg)**	**neisserial antigen immunization dose (µg)**	**PHB beads immunization dose (mg)**
NadA-PhaC	0.254	2	10
GNA2091-fHbp-G1-PhaC	0.677	7	10
PhaC-fHbp-G1-G1	0.110	1	10
**PHB beads**	**PhaC/ wet Bead (µg/mg)**	**PhaC immunization dose (µg)**	**PHB beads immunization dose (mg)**
PhaC, (non-antigen displaying)	0.977	7	7

Supplementary Table 5:

Carbohydrate/protein ratios and carbohydrate yield after conjugation and purification.

Conjugated	Carbohydrate/protein ratio (mg/mg)	Carbohydrate yield (%)
MenC-NadA-PhaC beads	0.20/1	4.0
MenC-PhaC beads	0.27/1	4.4
MenC-NadA-His6	2.4/1	30.2
MenC-DT	2.5/1	36.0

Supplementary Table 6

Conjugation site analysis results by liquid chromatography-coupled tandem mass spectrometry (LC-MS/MS). Tryptic and chymotryptic digests of non-conjugated and conjugated proteins either soluble or attached to PHB beads were analysed by liquid chromatography-coupled LTQ-Orbitrap tandem mass spectrometry. Significant differences in peptide abundances between the non-conjugated and conjugated proteins were identified by comparing precursor peak intensities using the SIEVE software (ThermoScientific).

**Samples**	**Chymotryptic (C)/tryptic (T) peptides >3-fold more abundant in non-conjugated sample versus conjugated sample based on LC/MS-MS analysis***	**Amino acid sequence of targeted protein showing putative conjugations sites in bold**
PhaC versus conjugated PhaC displayed on PHB beads (C)	NARALTELADAVEADAKTRQRIRF<br>GNARYRAIEPAPGRYVKAKA<br>TGKGAAASTQEGKSQPF+Carbamidomethyl (13) Formyl (3)<br>AAIRAIEVARDISGQDKINVL<br>RAIEPAPGRYVKAKA	PhaC (64.2kDa)<br><br>MATG**K**GAAASTQEG**K**SQPF<br>KVTPGPFDPATWLEWSRQW<br>QGTEGNGHAAASGIPGLDAL<br>AGVKIAPAQLGDIQQRYMKD<br>FSALWQAMAEGKAEATGPL<br>HDRRFAGDAWRTNLPYRFA<br>AAFYLLNARALTELADAVEA<br>DA**K**TRQRIRFAISQWVDAMS<br>PANFLATNPEAQRLLIESGGE<br>SLRAGVRNMMEDLTRGKISQ<br>TDESAFEVGRNVAVTEGAVV<br>FENEYFQLLQYKPLTDKVHA<br>RPLLMVPPCINKYYILDLQPE<br>SSLVRHVVEQGHTVFLVSWR<br>NPDASMAGSTWDDYIEHAAI<br>RAIEVARDISGQD**K**INVLGFC<br>VGGTIVSTALAVLAARGEHP<br>AASVTLLTTLLDFADTGILDV<br>FVDEGHVQLREATLGGGAG<br>APCALLRGLELANTFSFLRPN<br>DLVWNYVVDNYLKGNTPVP<br>FDLLFWNGDATNLPGPWYC<br>WYLRHTYLQNELKVPGKLT<br>VCGVPVDLASIDVPTYIYGSR<br>EDHIVPWTAAYASTALLANK<br>LRFVLGASGHIAGVINPPAKN<br>KRSHWTNDALPESPQQWLA<br>GAIEHHGSWWPDWTAWLAG<br>QAGAKRAAPANYGNARYRA<br>IEPAPGRYV**K**A**K**A

		Lysine residues (labelled bold) represent putative polysaccharide conjugations sites:<br>**K:** 5, 15, 139, 312, 498, 586, 588
NadA-PhaC versus conjugated NadA-PhaC displayed on PHB beads (C)	AAIRAIEVARDISGQDKINVL<br>ATSDDDVKKAATVAI<br>ATSDDDVKKAATVAIVAA<br>DVEADDFKGL<br>NARALTELADAVEADAK<br>NARALTELADAVEADAKTR<br>TKTVNENKQNVDAKVK	NadA (1-463)-PhaC (464-1051) fusion protein (112.8kDa)<br><br>MATSDDDV**KK**AATVAIVAA<br>YNNGQEINGFKAGETIYDIGE<br>DGTITQKDATAADVEADDF**K**<br>GLGLKKVVTNLT**K**TVNEN**K**<br>QNVDA**K**V**K**AAESEIEKLTTK<br>LADTDAALADTDAALDETTN<br>ALNKLGENITTFAEETKTNIV<br>KIDEKLEAVADTVDKHAEAF<br>NDIADSLDETNTKADEAVKT<br>ANEAKQTAEETKQNVDAKV<br>KAAETAAGKAEAAAGTANT<br>AADKAEAVAAKVTDIKADIA<br>TNKADIAKNSARIDSLDKNV<br>ANLRKETRQGLAEQAALSGL<br>FQPYNVGRFNVTAAVGGYKS<br>ESAVAIGTGFRFTENFAAKAG<br>VAVGTSSGSSAAYHVGVNYE<br>WKAGETIYDIGEDGTITQKD<br>KAGETIYDIGEDGTITQKDKA<br>GETIYDIGEDGTITQKDTSDD<br>DVKKAATVAIVTSDDDVKK<br>AATVAIVT**K**TVNEN**K**QNVD<br>A**K**V**K**AT**K**TVNEN**K**QNVDA<br>**K**V**K**ATSATGKGAAASTQEG<br>KSQPFKVTPGPFDPATWLEW<br>SRQWQGTEGNGHAAASGIPG<br>LDALAGVKIAPAQLGDIQQR<br>YMKDFSALWQAMAEGKAEA<br>TGPLHDRRFAGDAWRTNLPY<br>RFAAAFYLLNARALTELADA<br>VEADA**K**TRQRIRFAISQWVD<br>AMSPANFLATNPEAQRLLIES<br>GGESLRAGVRNMMEDLTRG<br>KISQTDESAFEVGRNVAVTE<br>GAVVFENEYFQLLQYKPLTD<br>KVHARPLLMVPPCINKYYILD<br>LQPESSLVRHVVEQGHTVFL<br>VSWRNPDASMAGSTWDDYI<br>EHAAIRAIEVARDISGQD**K**IN<br>VLGFCVGGTIVSTALAVLAA<br>RGEHPAASVTLLTTLLDFADT

		GILDVFVDEGHVQLREATLG<br>GGAGAPCALLRGLELANTFS<br>FLRPNDLVWNYVVDNYLKG<br>NTPVPFDLLFWNGDATNLPG<br>PWYCWYLRHTYLQNELKVP<br>GKLTVCGVPVDLASIDVPTYI<br>YGSREDHIVPWTAAYASTAL<br>LANKLRFVLGASGHIAGVINP<br>PAKNKRSHWTNDALPESPQQ<br>WLAGAIEHHGSWWPDWTA<br>WLAGQAGAKRAAPANYGNA<br>RYRAIEPAPGRYVKAKA<br>Lysine residues (labelled in bold) represent putative polysaccharide conjugations sites:<br>**K**: 9, 10, *60*, *73*, 79, 85, *87*, 429. 435, 441, *443*, *446*, *452*, *458*, *460*, 601 (139 in PhaC), 774 (312 in PhaC)<br>Italics: Conjugation sites not found in soluble NadA
NadA-PhaC versus conjugated NadA-PhaC displayed on PHB beads (T)	AATVAIVAAYNNGQEINGFK+Deamidated (11) Deamidated (12)<br>AATVAIVAAYNNGQEINGFK+Deamidated (12)<br>AATVAIVAAYNNGQEINGFK+Deamidated (12) Deamidated (17)<br>ASMAGSTWDDYIEHAAIR<br>CVGGTIVSTALAVLAAR+Carbamidomethyl (1)<br>DASMAGSTWDDYIEHAAIR<br>DFSALWQAMAEGK<br>DFSALWQAMAEGK+Carbamidomethyl (13)<br>DFSALWQAMAEGKAEATGPLHDR<br>EDHIVPWTAAYASTALLAN<br>EDHIVPWTAAYASTALLANK<br>EDHIVPWTAAYASTALLANKLR<br>EDHIVPWTAAYASTALLANKLR+Carbamidomethyl (20)<br>FAISQWVDAMSPANFLATNPEAQR+Oxidation (6)<br>FAISQWVDAMSPANFLATNPEAQR+Oxidation (6) Oxidation (10)<br>GLELANTFSFLR<br>GVPVDLASIDVPTYIYGSR<br>LADTDAALADTDAALDETTNALNK	NadA (1-463)-PhaC (464-1051) fusion protein (112.8kDa)<br><br>MATSDDDVKKAATVAIVAA<br>YNNGQEINGF**K**AGETIYDIGE<br>DGTITQKDATAADVEADDFK<br>GLGLKKVVTNLTKTVNENK<br>QNVDAKVKAAESEIEKLTTK<br>LADTDAALADTDAALDETTN<br>ALN**K**LGENITTFAEETKTNIV<br>KIDEKLEAVADTVDKHAEAF<br>NDIADSLDETNT**K**ADEAVKT<br>ANEA**K**QTAEETKQNVDAKV<br>KAAETAAGKAEAAAGTANT<br>AADKAEAVAAKVTDIKADIA<br>TNKADIAKNSARIDSLDKNV<br>ANLRKETRQGLAEQAALSGL<br>FQPYNVGRFNVTAAVGGYKS<br>ESAVAIGTGFRFTENFAAKAG<br>VAVGTSSGSSAAYHVGVNYE<br>WKAGETIYDIGEDGTITQKD<br>KAGETIYDIGEDGTITQKDKA<br>GETIYDIGEDGTITQKDTSDD<br>DVKKAATVAIVTSDDDVKK<br>AATVAIVTKTVNENKQNVDA<br>KVKATKTVNENKQNVDAKV<br>KATSATGKGAAASTQEGKSQ<br>PFKVTPGPFDPATWLEWSRQ

	LADTDAALADTDAALDETTNALN<br>K+Deamidated (23)<br>LDETNTKADEAVKTANEAK+Carb<br>amidomethyl (13) Deamidated (5)<br>Formyl (19)<br>NMMEDLTRGKISQTDESAFEVGR<br>PATWLEWSR<br>PDASMAGSTWDDYIEHAAIR<br>PNDLVWNYVVDNYLK<br>TDAALADTDAALDETTNALNK<br>VTPGPFDPATWLEWSR<br>VTPGPFDPATWLEWSR+Oxidation<br>(11)	WQGTEGNGHAAASGIPGLDA<br>LAGVKIAPAQLGDIQQRYMK<br>DFSALWQAMAEG**K**AEATGP<br>LHDRRFAGDAWRTNLPYRFA<br>AAFYLLNARALTELADAVEA<br>DAKTRQRIRFAISQWVDAMS<br>PANFLATNPEAQRLLIESGGE<br>SLRAGVRNMMEDLTRGKISQ<br>TDESAFEVGRNVAVTEGAVV<br>FENEYFQLLQYKPLTDKVHA<br>RPLLMVPPCINKYYILDLQPE<br>SSLVRHVVEQGHTVFLVSWR<br>NPDASMAGSTWDDYIEHAAI<br>RAIEVARDISGQDKINVLGFC<br>VGGTIVSTALAVLAARGEHP<br>AASVTLLTTLLDFADTGILDV<br>FVDEGHVQLREATLGGGAG<br>APCALLRGLELANTFSFLRPN<br>DLVWNYVVDNYL**K**GNTPVP<br>FDLLFWNGDATNLPGPWYC<br>WYLRHTYLQNELKVPGKLT<br>VCGVPVDLASIDVPTYIYGSR<br>EDHIVPWTAAYASTALLAN**K**<br>LRFVLGASGHIAGVINPPAKN<br>KRSHWTNDALPESPQQWLA<br>GAIEHHGSWWPDWTAWLAG<br>QAGAKRAAPANYGNARYRA<br>IEPAPGRYVKAKA<br>Red: Potential polysaccharide conjugation sites sterically impacting on trypsin digest (sites are not part of identified peptide)<br>K: 482, 601, 774, 913, 917<br>Lysine residues (labelled in bold) represent putative polysaccharide conjugations sites:<br>**K**: 30, 123, 173, 185, 552 (90 in PhaC), 875 (413 in PhaC), 960 (498 in PhaC)
Soluble NadA versus conjugated soluble NadA (T)	AAETAAGKAEAAAGTAN<br>AAETAAGKAEAAAGTANTAADK<br>AEAVAAK<br>AATVAIVAAYNNGQEINGFK<br>AATVAIVAAYNNGQEINGFK+Dea<br>midated (11)<br>AATVAIVAAYNNGQEINGFK+Dea<br>midated (12) Deamidated (17)<br>AATVAIVAAYNNGQEINGFK+Dea<br>midated(14)	NadA-His6: (49.2 kDa)<br>MATSDDDV**KK**AATVAIVAA<br>YNNGQEINGF**K**AGETIYDIGE<br>DGTITQ**K**DATAADVEADDFK<br>GLGLKKVVTNLTKTVNEN**K**<br>QNVDA**K**VKAAESEIEKLTTK<br>LADTDAALADTDAALDETTN<br>ALN**K**LGENITTFAEET**K**TNIV<br>**K**IDEKLEAVADTVD**K**HAEAF<br>NDIADSLDETNT**K**ADEAV**K**T<br>ANEA**K**QTAEETKQNVDAKV

	AGETIYDIGEDGTITQKDTSDDDV<br>KK<br>AGETIYDIGEDGTITQKDTSDDDV<br>KK+Deamidated (16)<br>AVGTSSGSSAAYHVGVNYEWK<br>HAEAFNDIADSLDETNTK<br>HAEAFNDIADSLDETNTKADEAV<br>K+Deamidated (16)<br>HAEAFNDIADSLDETNTKADEAV<br>K+Deamidated (6)<br>LADTDAALADTDAALDETTNALN<br>K<br>LADTDAALADTDAALDETTNALN<br>K+Deamidated (20)<br>LDETNTKADEAVKTANEAK+Carb<br>amidomethyl (13) Deamidated (5)<br>Formyl (19)<br>LEAVADTVDKHAEAFNDIADSLD<br>ETNTK<br>LGENITTFAEE<br>LGENITTFAEETKTNIVK<br>NITTFAEETK<br>SAAYHVGVNYEWK<br>SGSSAAYHVGVNYEWK<br>SSGSSAAYHVGVNYEWK<br>TDAALADTDAALDETTNALNK<br>TIYDIGEDGTITQK<br>TVAIVAAYNNGQEINGFK+<br>Deamidated (10) Deamidated (15)<br>TVAIVTKTVNENKQNVDAK	KAAETAAG**K**AEAAAGTANT<br>AAD**K**AEAVAA**K**VTDIKADIA<br>TNKADIAKNSARIDSLDKNV<br>ANLRKETRQGLAEQAALSGL<br>FQPYNVGRFNVTAAVGGYKS<br>ESAVAIGTGFRFTENFAAKAG<br>VAVGTSSGSSAAYHVGVNYE<br>W**K**AGETIYDIGEDGTITQ**K**D<br>KAGETIYDIGEDGTITQ**K**DKA<br>GETIYDIGEDGTITQ**K**DTSDD<br>DV**KK**AATVAIVTSDDDVKK<br>AATVAIVT**K**TVNEN**K**QNVD<br>A**K**VKATKTVNENKQNVDAK<br>VKAHHHHHH<br><br>Lysine residues (labelled in bold) represent putative polysaccharide conjugations sites:<br>**K: 9**, **10**, **30**, 47, **79**, **85**, **123**, 136, 141, 155, **173**, 179, **185**, 208, 223, 341, 358, 377, 396, 404, 405, **429**, **435**, **441** (bold number show sites identified in the NadA when fused to PhaC)

*, Samples were prepared as previously described[7]

Supplementary Table 7:

IgG/IgM ratio after first (1D) and third (3D) blood collection assayed against MenC.

Vaccinated groups	Mean IgG (1D)	Mean IgG (3D)	Mean IgM (1D)	Mean IgM (3D)	Ratio IgG/IgM (1D)	Ratio IgG/IgM (3D)
MenC-PhaC-NadA	231	3400	325	400	0.7	8.5
MenC-PhaC	200	5600	400	400	0.5	14.0
MenC-NadA-His6	125	2400	500	325	0.25	7.3
MenC-DT	100	1800	400	400	0.25	4.5

Supplementary Table 8

Size distribution of PHB beads in vaccine formulations (µm) as measured my dynamic laser scattering. Dx represents the particle diameter corresponding to X% cumulative particle size distribution.

Samples	PHB bead in PBS 1X			PHB bead in Al $(OH)_3$		
	Dx (10)	Dx (50)	Dx (90)	Dx (10)	Dx (50)	Dx (90)
NadA-PhaC beads	0.6	6.55	60.9	0.832	7.38	50.4
GNA2091-fHbp-G1-PhaC beads	3.8	9.5	68.6	3.7	9.8	70.6
PhaC-fHbp-G1-G1 beads	0.4	3.87	41.9	0.438	4.22	58.6
PhaC wild type bead	0.4	1.2	6.99	0.434	2.77	18.4

References

1 González-Miro, M. *et al.* Self-assembled particulate PsaA as vaccine against Streptococcus pneumoniae infection. *Heliyon* **3**, e00291 (2017).

2 Jones, C. & Lemercinier, X. Use and validation of NMR assays for the identity and O-acetyl content of capsular polysaccharides from Neisseria meningitidis used in vaccine manufacture. *Journal of pharmaceutical and biomedical analysis* **30**, 1233-1247 (2002).

3 Grage, K. *et al.* Bacterial polyhydroxyalkanoate granules: biogenesis, structure, and potential use as nano-/micro-beads in biotechnological and biomedical applications. *Biomacromolecules* **10**, 660-669 (2009).

4 Peters, V. & Rehm, B. H. In vivo monitoring of PHA granule formation using GFP-labeled PHA synthases. *FEMS Microbiol Lett* **248**, 93-100, doi:10.1016/j.femsle.2005.05.027 (2005).

5 Amara, A. A. & Rehm, B. H. Replacement of the catalytic nucleophile cysteine-296 by serine in class II polyhydroxyalkanoate synthase from Pseudomonas aeruginosa-mediated synthesis of a new polyester: identification of catalytic residues. *Biochem J* **374**, 413-421, doi:10.1042/BJ20030431 (2003).

6 Hooks, D. O., Blatchford, P. A. & Rehm, B. H. Bioengineering of bacterial polymer inclusions catalyzing the synthesis of N-acetylneuraminic acid. *Applied and environmental microbiology* **79**, 3116-3121 (2013).

7 Hooks, D. O. & Rehm, B. H. Insights into the surface topology of polyhydroxyalkanoate synthase: self-assembly of functionalized inclusions. *Applied microbiology and biotechnology* **99**, 8045-8053 (2015).
